# Muscle stem cells produce a protective Fibrillin-1 matrix to prevent precocious activation

**DOI:** 10.1126/sciadv.aeb8190

**Published:** 2026-10-16

**Authors:** Eleni Chrysostomou, Oussama Smail, Artemis Vlachopoulou, Takuto Hayashi, Nicolas Tcheng, Nicol Voermans, Dieter P. Reinhardt, Frederic Relaix, Philippos Mourikis

**Affiliations:** ^1^ Université Paris Cité, INSERM U1151, CNRS UMR8253, Institut Necker Enfants Malades (INEM), 75015 Paris, France.; ^2^ Univ Paris Est Créteil, INSERM, IMRB, EnvA, APHP, EFS, F-94010 Créteil, France.; ^3^ Faculté de Médecine Paris Descartes, 15 rue de l’École de médecine, 75006 Paris, France.; ^4^ Department of Neurology, Donders Institute for Brain, Cognition and Behaviour, Radboud University Medical Centre, Nijmegen, Netherlands.; ^5^ Faculty of Medicine and Health Sciences, Department of Anatomy and Cell Biology, McGill University, Montréal, QC, Canada.; ^6^ Faculty of Dental Medicine and Oral Health Sciences, McGill University, Montréal, Canada.

## Abstract

Diverse biological mechanisms regulate muscle stem cell quiescence, including inhibition of differentiation, adhesion, and translational control. Here, we identify the matrix glycoprotein Fibrillin-1 (FBN1) as a Notch-regulated, cell-autonomous effector, essential for maintaining quiescence. Known for its causal role in Marfan syndrome (MFS), our work positions FBN1 as a critical niche component that protects muscle stem cells from aberrant growth factor signaling. Targeted deletion of *Fbn1* in muscle stem cells leads to dose-dependent quiescence defects, characterized by loss of cellular projections, depletion of the stem cell pool, and progressive decline in muscle function. Consistently, human MFS muscle biopsies show abnormally activated satellite cells, implicating stem cell imbalance in the development of MFS-associated myopathy. Mechanistically, the loss of FBN1 up-regulates transforming growth factor–β (TGFβ) signaling, and pharmacological blockade of TGFβ signaling rescues the cellular and physiological defects of FBN1-deficient muscle. These findings reveal a new quiescence-preserving mechanism through extracellular matrix–mediated shielding from mitogenic signals and position stem cell dysfunction as a driver of MFS myopathy.

## INTRODUCTION

The niche of muscle stem cells, also known as satellite cells, provides both structural and signaling cues that preserve stem cell quiescence, a reversible cell cycle arrest crucial for maintaining long-term regenerative potential (*1*). Among the core regulators of quiescence is the Notch signaling pathway, which globally operates by suppressing activation/differentiation programs and preserving stemness. In skeletal muscle, Notch exerts its function through multiple effectors: it blocks myogenic gene expression via the *Hes*/*Hey* bHLH repressors, regulates microRNAs that ensure adhesion and anchorage, and induces the expression of extracellular matrix (ECM) collagen type V, which acts as a MuSC-derived ligand for the Calcitonin receptor (*2*–*5*). Here, we extend this framework by identifying Fibrillin-1 (FBN1), a ubiquitous ECM glycoprotein known for its structural role and regulatory influence on growth factor signaling, as a direct target of the Notch pathway.

In the ECM, FBN1 polymerizes into microfibrils, providing essential architectural and regulatory functions in elastic tissues. Microfibrils are also found in elastin-free cellular environments, where they form interfaces with basement membranes (*6*, *7*). FBN1 is a large, cysteine-rich extracellular glycoprotein that contains seven TB [transforming growth factor–β (TGFβ)–binding protein-like] domains and two hybrid domains, the latter combining structural features of calcium-binding epidermal growth factor (EGF)–like (cbEGF) and TB domains (*8*). Mutations in *FBN1* underlie Marfan syndrome (MFS), an autosomal dominant connective tissue disorder with a reported incidence of 1 in 3000 to 5000 individuals. These mutations are broadly classified as haploinsufficiency, which reduce functional protein levels, or dominant-negative, where mutant FBN1 incorporates into microfibrils and interferes with their function. The variant type, however, does not reliably predict disease severity and should be interpreted with caution in clinical counseling (*9*, *10*). These mutations act either hypomorphic or dominant-negative and lead to defective mechanosensing of the cellular microenvironment and to elevated TGFβ signaling, a hallmark of MFS pathology (*11*, *12*). FBN1-deficient mice (*Fbn1*^−/−^) die within the first 2 weeks of postnatal life due to ruptured aortic aneurysm, impaired pulmonary function, and/or diaphragmatic collapse (*13*). In skeletal muscle, mice carrying one *Fbn1^C1041G^* allele, a missense mutation modeled after a mutation commonly found in patients with MFS, exhibit impaired tissue regeneration (*14*).

Pharmacological inhibition of TGFβ signaling has been widely explored as a therapeutic strategy in MFS and other myopathies. Among these, losartan potassium, a Food and Drug Administration (FDA)–approved angiotensin II receptor type 1 (AT1) antagonist, has emerged as a particularly effective candidate. Originally developed as an antihypertensive, losartan was shown to reduce TGFβ signaling, thereby indirectly antagonizing downstream fibrotic and inflammatory responses (*15*). In MFS mouse models, losartan prevents aortic root dilation, delays aneurysm progression, and improves cardiac, skeletal, and pulmonary phenotypes, making it one of the most extensively studied nonsurgical therapies for the disease (*14*, *16*). In skeletal muscle, losartan enhances tissue regeneration in both *Fbn1*-mutant and Dystrophin-deficient (*mdx*) mice, where it mitigates fibrosis, preserves myofiber integrity, and improves muscle function (*14*). To date, these effects are attributed to its capacity to create a more permissive environment for muscle stem cell–mediated repair (*14*, *17*, *18*).

Despite these results and the clear evidence of muscle atrophy in patients with MFS, the direct role of FBN1 on satellite cell maintenance had not been previously investigated. Our study fills this gap, uncovering a Notch-driven, cell-autonomous function for FBN1 in maintaining stem cell quiescence and protecting against aberrant growth factor signaling. We further demonstrate that pharmacological inhibition of TGFβ not only rescues stem cell numbers and quiescence morphology in *Fbn1* cKO mice but also restores muscle performance and tissue integrity, suggesting stem cell dysfunction as a contributing source of the pathology. These findings position losartan as a promising therapy for MFS-associated myopathy and provide a mechanistic basis for its beneficial effects beyond vascular tissues.

## RESULTS

### Identification of FBN1 as a potential regulator of quiescence in skeletal muscle stem cells

Notch signaling is essential for satellite cell maintenance, acting through a diverse range of targets (*2*). To identify additional Notch-regulated and ECM-associated regulators of satellite cell quiescence, we mined ChIP sequencing data for intracellular Notch1 (NICD) and its downstream effector RBPJ in myogenic cells and integrated them with transcriptomic profiles of in situ fixed quiescent satellite cells (qSCs) (*19*–*21*). Among the genes showing both enhancer occupancy by NICD/RBPJ and strong expression in qSCs, we identified *Fbn1*, which encodes a key ECM glycoprotein (Fig. 1A and fig. S1A).

**Fig. 1.**
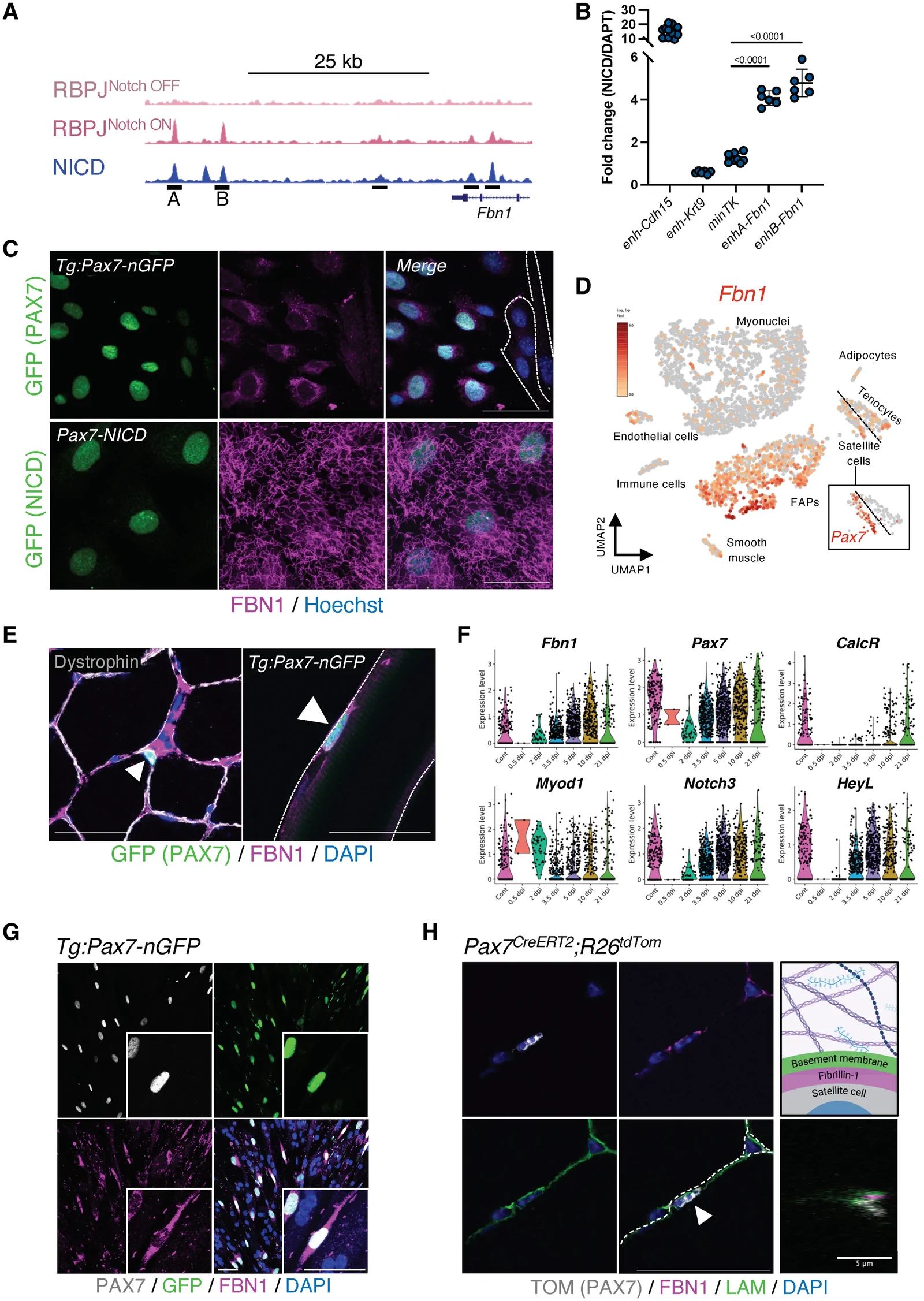
FBN1 is a Notch-regulated ECM component of qSCs. (**A**) ChIP sequencing for RBPJ and nuclear Notch1 (NICD) on mouse myogenic C2C12 cells indicating enhancers associated with *Fbn1* locus (black rectangles); enhancers A and B were used for luciferase activity assays. (**B**) Luciferase-based assay on cloned enhancers in C2C12 cells transfected with NICD (Notch On) or treated with the γ-secretase inhibitor DAPT (Notch Off). Cadherin 15 (Cdh15) enhancer used as a positive control, Keratin 10 (Krt19) enhancer as a negative control, and minTK promoter as a baseline control. (**C**) Isolated satellite cells from Control (*Tg:Pax7-nGFP*) and Notch-overexpressing (*Pax7^CreERT2^; R26^stop-NICD^*) mice (*n* = 3 per genotype). All NICD+ cells appeared larger and produced excessive FBN1. Dashed lines indicate differentiated PAX7^Low^/FBN1^−^ cells. (**D**) UMAP of single-nucleus RNA-seq from resting; adult TA muscles [Machado *et al.* (*19*)] indicating the expression of *Fbn1*. The inset shows *Pax7* expression in the satellite cell cluster. (**E**) Immunofluorescence for FBN1 expression in the interstitial space (TA muscle sections; left) and in satellite cells of isolated myofibers (100% GFP^+^/FBN1^+^, *Tg:Pax7-nGFP* mice, EDL muscle; *n* = 10 to 15 fibers per mouse, three mice; right). DAPI, 4′,6-diamidino-2-phenylindole. (**F**) Transcriptomic data [scRNA-seq; Oprescu *et al.* (*23*)] on resting (Control) and regenerating muscles [0.5, 2, 3.5, 5, 10, and 21 days postinjury (dpi)] showing the expression dynamics of *Fbn1* relative to *Pax7*, *CalcR*, *Myod1*, and Notch pathway members *Notch3* and *HeyL*. (**G**) High FBN1 expression in quiescent-like “reserve” cells (PAX7^+^) generated from FACS-isolated satellite cells (*n* = 3 mice). (**H**) High-resolution imaging of TA muscle sections of FBN1 in relation to the basal lamina marked by Laminin (LAM). Orthogonal view and schematic representation on the right-hand side panels. Arrowhead points to a satellite cell. Unpaired one-way ANOVA ± SD; scale bars, 40 μm.

Two putative enhancer elements of *Fbn1* were cloned and showed a four- to sixfold increase in luciferase assays upon Notch stimulation (Fig. 1B). Moreover, when we isolated and cultured satellite cells from mice conditionally overexpressing NICD in satellite cells (Pax7-NICD for *Pax7^CreERT2^; R26^stop-NICD^*), we observed a notable induction of extracellular FBN1 compared to control cells (Fig. 1C). To assess the functional role of this protein, we knocked down *Fbn1* using small interfering RNA (siRNA) in quiescent-like cells generated in cell culture. For that, Pax7-NICD satellite cells were isolated and cultured to confluency; sustained Notch activation prevents differentiation and fusion, leading instead to contact inhibition and subsequent cell cycle exit (Pax7^+^/EdU^−^ cells). Upon *Fbn1* knockdown, however, cells failed to maintain quiescence and prematurely entered the cell cycle, as evidenced by an increased proportion of PAX7^+^/EdU^+^ cells (fig. S1, B and C).

Single-nucleus RNA sequencing (RNA-seq) data on resting tibialis anterior (TA) muscles from adult mice revealed that *Fbn1* is expressed by multiple cell types in skeletal muscle, with the highest levels detected in mesenchymal fibro-adipogenic progenitors (FAPs) and robust expression also observed in qSCs (Fig. 1D). To confirm expression at the protein level, we performed immunofluorescence analysis in TA hindlimb muscle sections using a specific antibody raised against mouse FBN1 (*22*). FBN1 was detected in the interstitial space, consistent with expression by FAPs, and showed complete colocalization with green fluorescent protein (GFP) in satellite cells on isolated myofibers from *Tg:Pax7-nGFP* mice (Fig. 1E). To assess regulation of *Fbn1* in satellite cells, we analyzed bulk RNA-seq data from Machado *et al.* (*19*), which showed a significant reduction in expression following quiescence exit, with a 2.5-fold decrease after 120 min of activation (*P* = 5 × 10^−7^). This dynamic expression was further detected in single-cell RNA-seq (scRNA-seq) data from injured TA muscles, where *Fbn1* expression positively correlated with Notch signaling activity, including expression of *Notch3*, *HeyL*, the satellite cell and myoblast marker *Pax7*, and the quiescence marker *CalcR*, but negatively correlated with the activation marker *Myod1*, consistent with the known repression of *Myod1* by Notch signaling [Fig. 1F, scRNA-seq from (*23*); fig. S1D, *Fbn1*/*HeyL* correlation in qSCs]. Moreover, FBN1 protein correlated with PAX7 in cultured cells, whereas expression was absent in differentiated myonuclei (Fig. 1G and fig. S1E). Last, to assess the regionalization of FBN1 in relation to the satellite cell niche in vivo, we used high-resolution imaging coupled with single-molecule detection confocal microscopy to visualize FBN1- and Laminin- stained sections of TA muscles and found that FBN1 deposition is polarized in the intermediate niche, located between the basement membrane and the satellite cell (Fig. 1H).

Together, these data indicate that Notch signaling–driven FBN1 is produced by qSCs and deposited at the interphase of satellite cells and the basement membrane. These features suggest that FBN1 may play a cell-autonomous role in maintaining satellite cell quiescence.

### FBN1 is a functional component of the quiescent niche

To determine whether FBN1 produced by satellite cells is a functional component of the niche, we generated mice with a conditional deletion of one or both *Fbn1* alleles upon tamoxifen administration in the Pax7^+^ satellite cells while simultaneously genetically marking them with the red fluorescent protein (RFP) tdTomato (*Pax7^CreERT2^;Fbn1^+/+^;R26^tdTom^*; *Pax7^CreERT2^;Fbn1^fl/+^;R26^tdTom^*; *Pax7^CreERT2^;Fbn1^fl/fl^;R26^tdTom^*, hereafter referred to as Control, cKO^Het^, and cKO^Homo^ mice, respectively). The fidelity of the mouse model was validated by scoring Pax7^+^/Tom^+^ cells (97%; fig. S2A), and the reduction of *Fbn1* levels was validated by reverse transcription quantitative polymerase chain reaction (RT-qPCR) (82.5% reduction; fig. S2B).

Short-term chase after deletion of *Fbn1* in satellite cells (cKO^Homo^ mice, 2 weeks post–tamoxifen treatment) resulted in their significant decrease in homeostatic muscle (47.7% reduction), demonstrating that continuous production of FBN1 by satellite cells is essential for quiescence maintenance (Fig. 2, A to C). The concomitant increase in TOM^+^ myofibers indicated that the *Fbn1* cKO cells underwent terminal differentiation and fused prematurely (Fig. 2D). Increased red fibers, but not significantly reduced satellite cells, were also scored at this time point in the cKO^Het^ mice (Fig. 2, C and D). When FBN1 was depleted for a longer period (4 months post–tamoxifen diet; Fig. 2A, bottom), the stem cell pool in cKO^Homo^ mice was almost completely exhausted (83.8% reduction; Fig. 2C). The *Fbn1* heterozygous mice also showed a significant reduction in satellite cells, alongside with the corresponding increase in TOM^+^ myofibers (Fig. 2, B to D). The drastic depletion of the satellite cell pool likely contributes to the global compromise of interstitial space architecture that we observed (Fig. 2E), in agreement with previous observations (*24*). To further characterize this intriguing phenotype in these muscles, we fluorescence-activated cell sorting (FACS) purified FAPs and assessed Notch signaling activity (*Hes1*, *Hey1*, *Hey2*, and *HeyL*), expression of ECM components (*Fbn1*, *Col1a1*, *Col5a1*, and *Col5a3*), basement membrane components (*Lama2* and *Col4a1*), and FAP markers (*Pdgfra* and *Dcn*) by RT-qPCR (fig. S2, C to J), without detecting any differences. Of note, the percentage of Sca1^+^/CD31^−^ FAPs in Control and cKO^Homo^ mice was comparable (fig. S2E). Although these aspects of FAPs seem unaffected, the overall defects in muscle architecture were associated with reduced treadmill endurance and grip strength (Fig. 2, F and G). Last, no statistically significant differences were observed at the fiber cross-sectional area (CSA) in both short-term and long-term *Fbn1* depletion cohorts, although cKO^Homo^ mice showed a tendency to have larger myofibers (fig. S2, K and L).

**Fig. 2.**
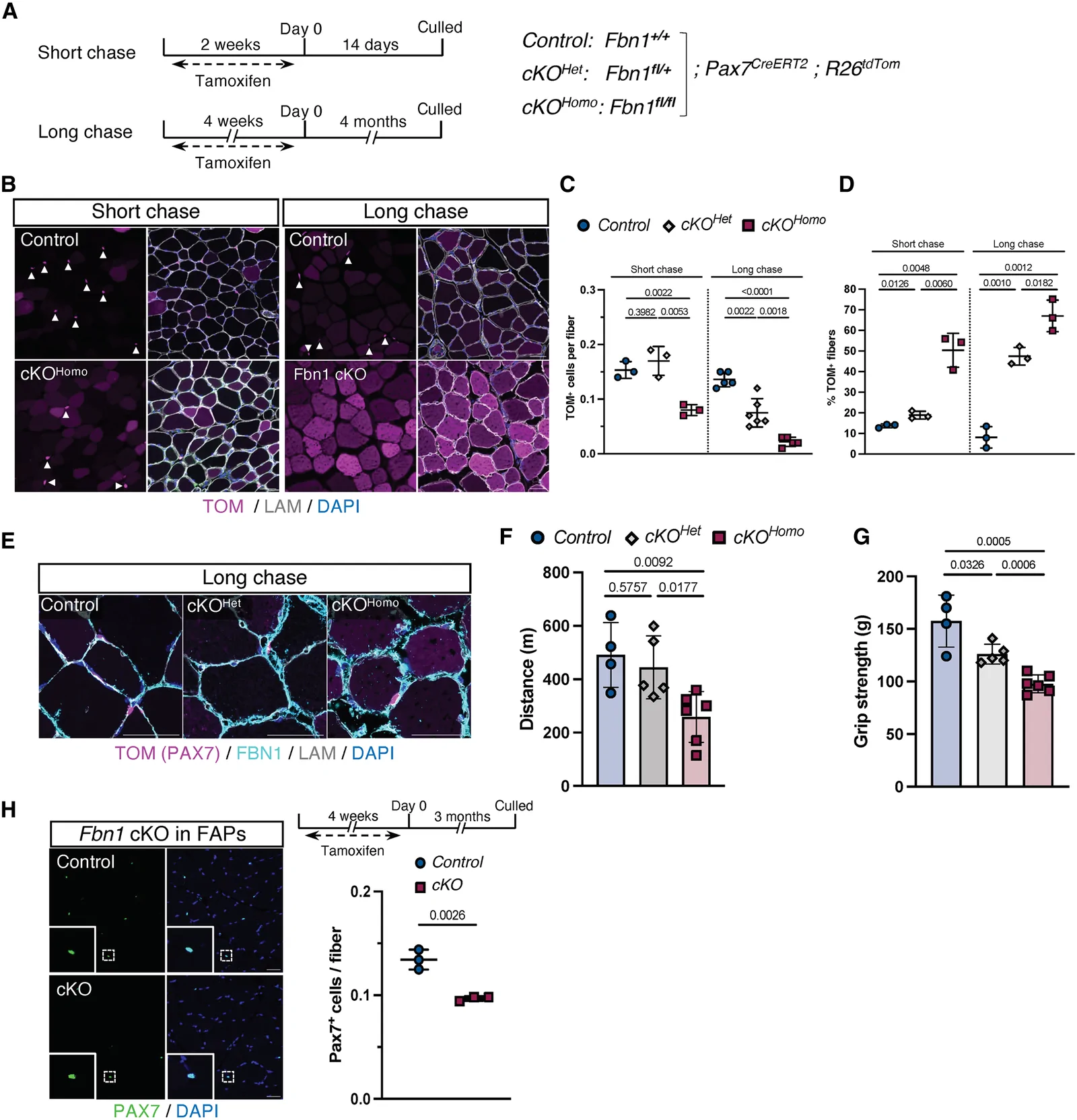
Satellite cell–derived FBN1 maintains quiescence and prevents premature fusion in vivo. (**A**) Experimental scheme for short (2 weeks post–tamoxifen treatment) and long (4 months post–tamoxifen treatment) chase in control (*Pax7^CreERT2^;Fbn1^+/+^;R26^tdTomato^*), *Fbn1* heterozygous (cKO^Het^: *Pax7^CreERT2^;Fbn1^fl/+^;R26^tdTomato^*), and *Fbn1* homozygous conditional knockout (cKO^Homo^: *Pax7^CreERT2^;Fbn1^fl/fl^;R26^tdTomato^*) satellite cells. (**B**) Immunostaining of TA muscle sections from Control and Fbn1 cKO^Homo^ mice from short and long chase after *Fbn1* deletion (*n* = 3 mice per genotype). (**C**) Satellite cell quantification in homeostatic TA muscles in a *Fbn1* allelic series (*n* = 3 to 6 mice per genotype). (**D**) Quantification of TOM^+^ fibers from homeostatic TA muscles in short-chased and long-chased animals (*n* = 3 mice per genotype). (**E**) Immunostaining of TA muscle sections from *Fbn1* long-chase depletion mice (*n* = 3 mice per genotype). (**F**) Quantification of treadmill performance analysis in mice from the long-term Fbn1 depletion cohort (*n* = 4 to 6 mice per genotype). (**G**) Quantification of grip strength performance from the long-term Fbn1 depletion cohort (*n* = 4 to 6 mice per genotype). (**H**) Immunostaining of TA muscles from Control (*Pdgfra^CreERT2^;Fbn1^+/+^;R26^tdTomato^*) and *Fbn1* cKO in FAPs (*Pdgfra^CreERT2^;Fbn1^fl/fl^;R26^tdTomato^*) mice from long chase after *Fbn1* deletion (*n* = 3 mice per genotype) and satellite cell quantification. One-way ANOVA ± SD [(C), (D), and (F) to (G)]; unpaired *t* test ± SD (H); scale bars, 40 μm.

In the adult mouse, *Pax7* is expressed in tissues beyond skeletal muscle, including a subset of undifferentiated spermatogonia, the central nervous system and in the pituitary gland, a key paracrine regulator (*25*, *26*). For that, we considered the possibility that systemic effects could arise from inadvertently targeting *Fbn1* in *Pax7*-expressing cells outside the muscle. To avoid misinterpreting the muscle phenotypes due to such paracrine effects in the *Pax7^CreERT2^; Fbn1^flox^* mice, we examined the pituitary gland where *Fbn1* is expressed. On the basis of published scRNA-seq data (*27*), *Pax7* and *Fbn1* are not coexpressed in the same cell populations (fig. S2M). To validate this in our model, we isolated the pituitary gland and performed immunostaining on sections for both PAX7 and FBN1. Consistent with the transcriptomic data, we observed no coexpression of the two proteins at the cellular level (fig. S2N). The involvement of testicular PAX7^+^ cells was excluded because we did not observe a sex bias.

To further investigate the influence of systemic *Fbn1* depletion on satellite cells, we selectively targeted *Fbn1^flox^* in mesenchymal cells. To achieve FBN1 knockdown, we generated compound mice carrying the *Pdgfra^CreERT2^* driver and the *Fbn1^flox^* allele (fig. S2O). Three months after tamoxifen administration, we observed a small reduction in the number of PAX7^+^ cells in the cKO muscles (Fig. 2H). It is important to note that *Pdgfra* is broadly expressed across multiple tissues, and *Pdgfra-Fbn1* cKO mice developed pathological phenotypes (e.g., abdominal hernias). Therefore, the observed decrease in satellite cell abundance could result from *Fbn1* loss specifically in FAPs but may also reflect broader systemic dysregulation or circulating signals triggered by widespread FBN1 deficiency.

### Loss of FBN1 leads to TGFβ activation of satellite cells

It has been shown that FBN1 can sequester latent TGFβ complexes in the ECM, and upon its destabilization, bioactive TGFβ is released from the ECM (*28*). Whether a similar mechanism operates in satellite cells remained unknown. Using pSMAD3 as a reporter of TGFβ signaling activity, we found cKO satellite cells with ectopic pathway activation. This response was FBN1 dose dependent with cKO^Het^ muscles exhibiting almost half the number of pSMAD3^+^ PAX7^+^ cells compared to cKO^Homo^ (Fig. 3, A and B). To determine whether satellite cell–derived FBN1 sequesters latent TGFβ, we performed an ex vivo enzyme-linked immunosorbent assay (ELISA)–based ligand depletion assay (Fig. 3C). Satellite cells from Control and *Fbn1* cKO muscles were cultured in TGFβ-containing medium, and total TGFβ levels were quantified after a short incubation period. Conditioned medium (CM) from control cells showed a clear reduction in TGFβ levels compared to the starting medium, whereas this decrease was not significant in CM from *Fbn1*-deficient satellite cells (Fig. 3D).

**Fig. 3.**
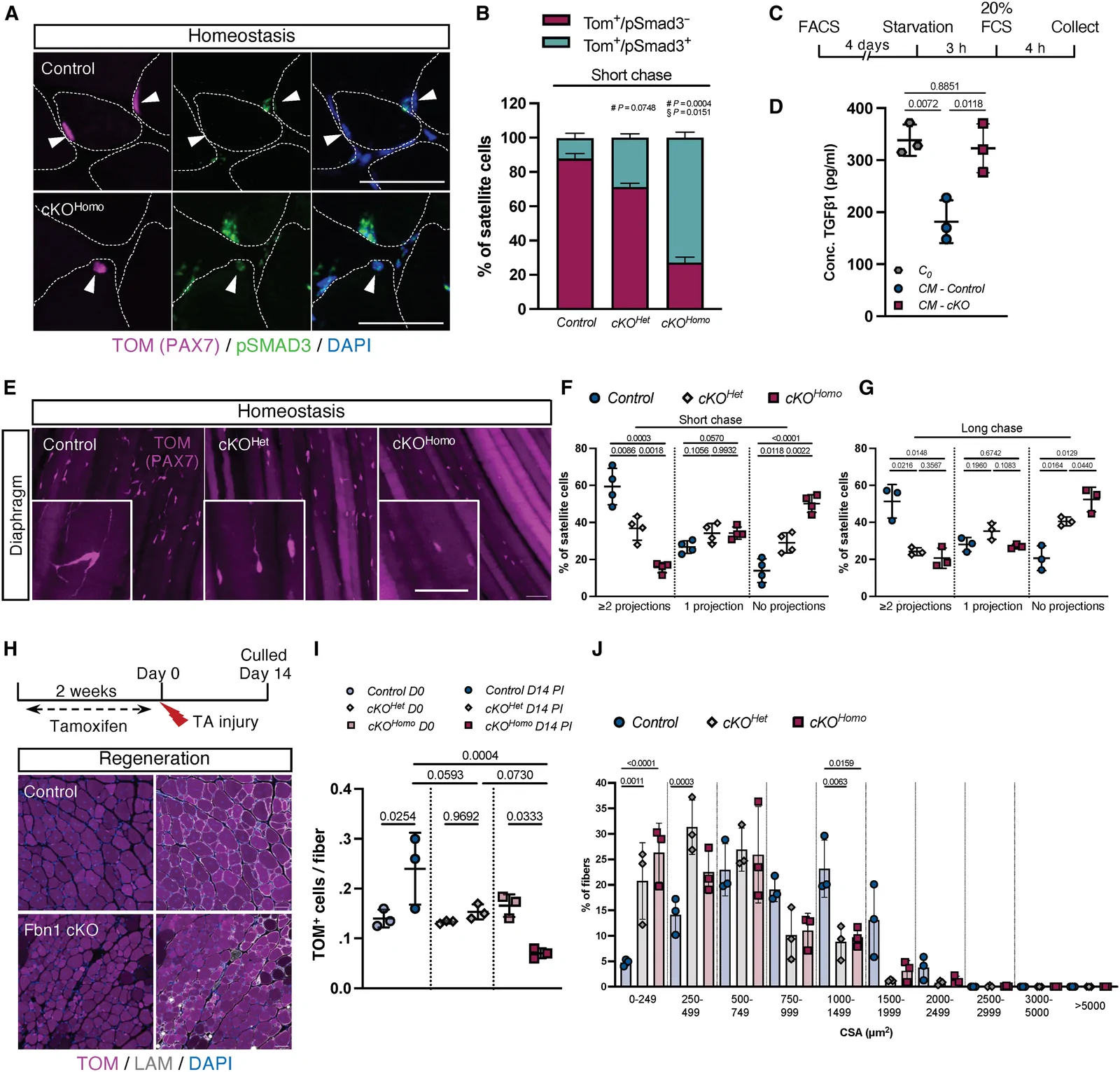
Decrease in FBN1 activates TGFβ signaling in satellite cells and disrupts quiescence projections. (**A**) Immunostaining of resting TA muscle sections from Control and *Fbn1* cKO^Homo^ TA muscles 2 weeks after tamoxifen-induced *Fbn1* deletion. Dashed lines outline the myofibers. Arrowheads point TOM^+^ satellite cells. (**B**) Quantification of Tom^+^/pSmad3^±^. (**C**) Experimental scheme of the ELISA-based ligand depletion assay for long chase (4 months post–tamoxifen treatment) control (*Pax7^CreERT2^;Fbn1^+/+^;R26^tdTomato^*) and *Fbn1* homozygous conditional knockout (cKO^Homo^: *Pax7^CreERT2^;Fbn1^fl/fl^;R26^tdTomato^*) satellite cells. h, hours. (**D**) Quantification of total TGFβ levels in starting medium (C_0_) and CM from Fbn1 control and cKO satellite cell cultures. (**E**) Immunostaining of diaphragm muscles from Control and cKO Fbn1 mice (short-term chase). Quantification of satellite cell projections divided into three groups: ≥2 projections, one projection, and no projections, (**F**) in short (2 weeks) and (**G**) in long (4 months) chase after *Fbn1* deletion. (**H**) Experimental scheme of muscle injury with BaCl_2_ and 14 days of regeneration. (**I**) Quantification of PAX7^+^ cells in TA muscle sections at 0 and 14 days postinjury (*n* = 3 mice per genotype). (**J**) Quantification of fiber CSA from the short-term, regenerative Fbn1 depletion cohort (*n* = 3 mice per genotype). One-way ANOVA ± SD [(B), (C), (F) to (G), (I) and (J)]; (B): # compared to Control; § compared to cKO^Het^; scale bars, 40 μm.

Unexpectedly, the mutant cells, despite being activated (pSMAD3^+^) and fusing (TOM^+^ fibers), bypassed the proliferation phase and directly underwent fusion while remaining Ki67^−^ (fig. S3A), a phenotype that, to date, has only been described in *Rbpj* mutant satellite cells (*3*, *29*). To further characterize the state of the *Fbn1* cKO satellite cells, we performed additional immunostaining for MyoD and phospho-S6 (pS6), a surrogate marker of mTORC1 activity and an established indicator of early satellite cell activation (*30*), along with pSmad3. As shown in fig. S3 (B and C), *Fbn1* cKO satellite cells are in an aberrant activation state compared to control satellite cells.

Given this atypical, early activation state, we scored for cytoplasmic projections, a hallmark of qSCs (*31*, *32*). For that, we examined the diaphragm, a thin, easily accessible muscle well suited for whole-mount imaging and rich in PAX7^+^ cells. As shown in Fig. 3 (E to G), most *Fbn1* cKO satellite cells had exited quiescence, as indicated by the attenuation of the quiescent projections. This response was also strictly dose dependent, with cKO^Het^ satellite cells exhibiting an intermediate phenotype between control and cKO^Homo^ cells. Moreover, the projections defect in *Fbn1* heterozygous cells gradually aggravated with time, confirming FBN1’s haploinsufficiency in the mouse.

We also examined the quiescent status of satellite cells in human biopsies from vastus lateralis muscles from healthy individuals and patients with MFS. Biopsies were obtained from two patients with MFS, a 58-year-old female and a 32-year-old male, as well as from age-matched and sex-matched healthy controls, a 54-year-old female and a 31-year-old male. The patients with MFS carried missense mutations in *FBN1*, located in exons 6 and 63, respectively (*33*). Consistent with our findings in mice, satellite cells from patients with MFS exhibited elevated levels of nuclear phosphorylated SMAD3 (pSMAD3) (fig. S3, D and E). Although both MFS samples showed increased pSMAD3 staining, the number of satellite cells appeared unchanged relative to controls (fig. S2F). Notably, *Fbn1^+/−^* mice also showed increased pSMAD3 in satellite cells, yet this was accompanied by a progressive, age-related decline in satellite cell number. One possible explanation for this discrepancy lies in the nature of the mutation as the mouse model carries a null allele of *Fbn1*, whereas the patients with MFS harbor heterozygous missense mutations, which may have different effects in satellite cells.

To investigate the regeneration and renewal potential of *Fbn1* cKO satellite cells, we performed BaCl_2_-mediated injuries of TA muscles and analyzed them 14 days later (Fig. 3H). Despite abundant FBN1 in regenerating muscle likely produced by resident fibroblasts (fig. S3G), fewer self-renewing PAX7^+^ cells were observed in the *Fbn1* cKO mice (Fig. 3I), confirming a cell-autonomous role for FBN1. In addition, *Fbn1* cKO satellite cells failed to return to quiescence during regeneration as indicated by the increased levels of pSMAD3 and, to our surprise, the proliferation levels scored by the Ki67 marker remained the same between control and cKO cells (fig. S3, H and I). Notably, mutant cells produced significantly smaller nascent myofibers compared to control cells (Fig. 3J). To investigate self-renewal in a more tractable and fibroblast-free system, we targeted *Fbn1* in satellite cells using siRNA on isolated myofibers in culture. Consistent with our in vivo observations, *Fbn1* knockdown resulted in a marked decrease in the number of the self-renewing PAX7^+^MYOD^−^ cells (10.5%), compared to scramble control (27.5%) (fig. S3, J to L).

### AT1 receptor antagonist restores quiescence of FBN1-deficient satellite cells and prevents muscle performance decline

Previous studies have shown that administration of losartan in multiple myopathic states can restore muscle architecture and prevent aortic aneurisms in MFS mouse models (*15*, *34*, *35*). Also, in both homeostatic and regenerating muscles, losartan has been shown to abrogate TGFβ signaling and restore muscle strength (*14*). However, the mechanisms underlying these effects are not well characterized, and the existing data on satellite cell behavior may be misleading due to the use of markers that are not broadly validated for distinguishing quiescence, activation, and differentiation in the muscle field (*14*).

Our genetic experiments demonstrated that targeted deletion of *Fbn1* in satellite cells leads to ectopic activation of TGFβ signaling, depletion of the stem cell pool, and severe impairment of muscle homeostasis and function. To test whether losartan could mitigate these effects, we administered the drug to both mutant and Control mice using short-term and long-term treatment regimens. We first validated efficient TGFβ signaling inhibition by losartan in satellite cells (fig. S4, A to C). Notably, the losartan-target receptor AT1 is not expressed in either qSCs or activated myoblasts (fig. S4D). This suggests that losartan’s inhibitory effect on TGFβ signaling is AT1 independent or results from systemic effects, such as a reduction in circulating TGFβ levels, as previously reported (*36*, *37*).

Treatment of *Fbn1* mutant mice with losartan for 4 weeks (short-term) prevented satellite cell loss (Fig. 4, A to C), whereas pSMAD3 in Pax7^+^ cells in both cKO^Het^ and cKO^Homo^ muscle was restored to levels comparable to Control-treated mice (fig. S4E). In parallel, we directly inhibited TGFβ signaling in vivo by administrating a neutralizing antibody against TGFβ1, TGFβ2, and TGFβ3 in Control and *Fbn1* cKO mice. As shown in fig. S4F, anti-TGFβ efficiently blocked ectopic pSmad3. This was accompanied by a robust rescue in the mutant satellite cell numbers, comparable to homeostatic control levels (Fig. 4, D and E).

**Fig. 4.**
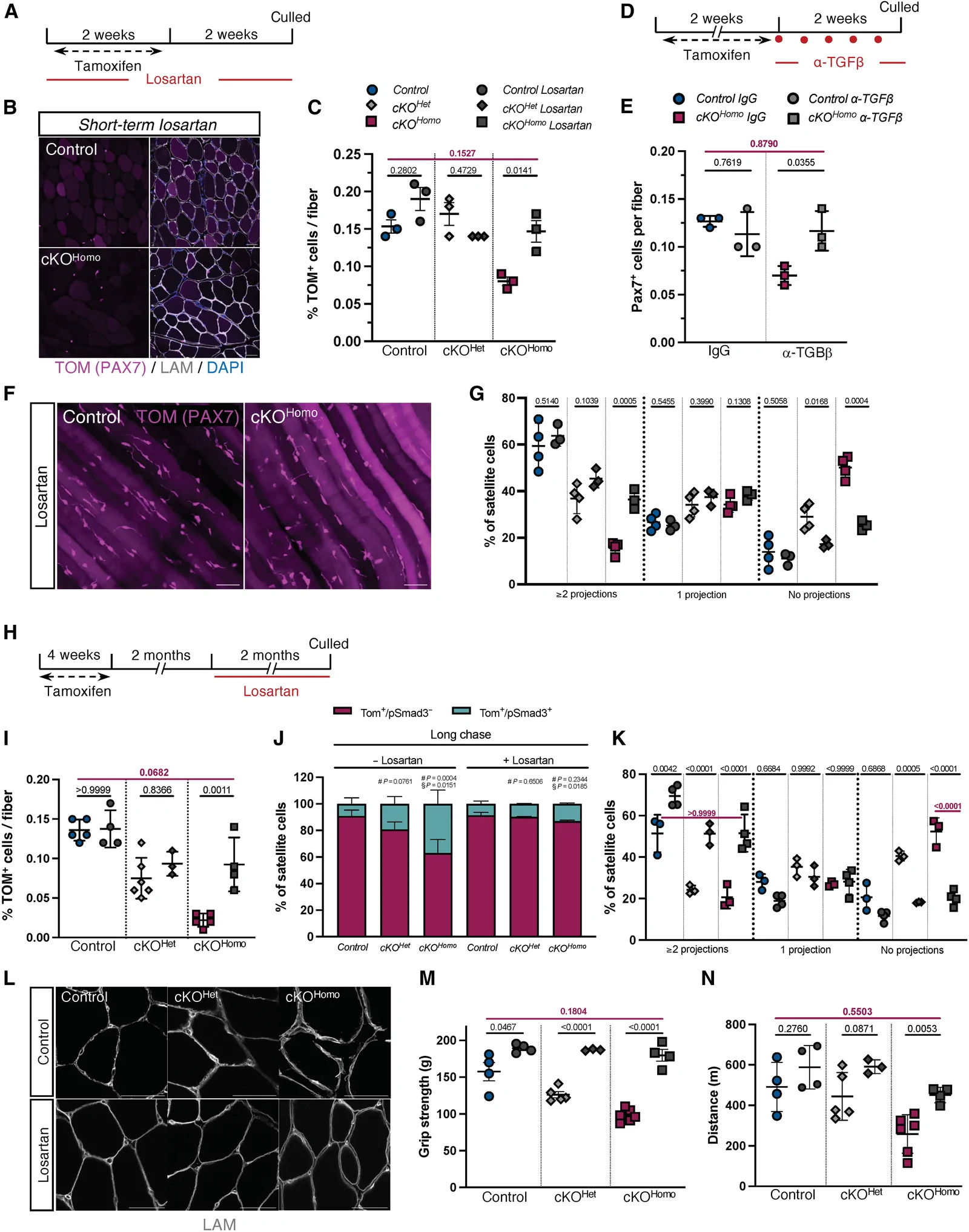
AT1 receptor antagonist restores quiescence of FBN1-deficient satellite cells and prevents muscle performance decline. (**A**) Experimental schematic of short-term treatment with losartan (4 weeks) in *Fbn1* cKO mice. (**B**) Immunostaining of Control and *Fbn1* cKO TA muscle sections from short-term losartan treatment (*n* = 3 mice per genotype) and (**C**) satellite cell quantification. (**D**) Experimental schematic of tamoxifen and anti-TGFβ administration in Control and *Fbn1* cKO mice and (**E**) satellite cell quantification. (**F**) Immunostaining of diaphragm muscles from Control and *Fbn1* cKO mice treated with losartan, short-term. (**G**) Quantification of satellite cell projections in mice treated with losartan, short-term. (**H**) Experimental schematic of long-term (2 months) treatment with losartan in *Fbn1* cKO mice, starting at 2 months after end of tamoxifen administration. (**I**) Satellite cell quantification and (**J**) Tom^+^/pSmad3^±^ in homeostatic TA muscles with and without losartan treatment (*n* = 3 to 6 mice per genotype per treatment). (**K**) Quantification of satellite cell projections in mice treated with losartan, long-term. (**L**) Immunostaining of TA muscle sections from Control and *Fbn1* cKO TA muscles from cohorts with and without losartan treatment. (**M**) Quantification of grip strength and (**N**) treadmill performance from the long-term *Fbn1* depletion cohort on losartan (*n* = 3 to 6 mice per genotype). Losartan: 0.6 g/liter in drinking water; neutralizing anti-TGFβ antibody or IgG control: 3.7 μg/g via intraperitoneal injection; one-way ANOVA ± SD [(B), (E), (G), (I) to (K), (M), and (N)]; # compared to Control; § compared to cKO^Het^; scale bars, 40 μm.

Induction of TGFβ signaling is linked to proliferating muscle cells (fig. S4, B and C), whereas the retraction of the quiescent projections is a hallmark of early activation, which occurs before proliferation (*31*, *32*, *38*). To measure the impact of losartan on satellite cell activation state, we scored the cytoplasmic projections and compared treated muscles to untreated. Losartan shifted the satellite cell population toward a quiescent state (≥2 projections) and away from an activated state (no projections) (Fig. 4, F and G). The shift to a quiescent-like morphology of satellite cell projections comparable to control levels was also observed when animals where administered the anti-TGFβ treatment (fig. S4G). No statistically significant differences were observed in the fiber CSA between genotypes (fig. S4H). Compared to the nontreated mice, losartan-treated myofibers fully shifted toward bigger CSA, possibly due to the inhibitory role of the TGFβ pathway on myocyte fusion (*38*). Losartan did not show any obvious signs of adverse effects on satellite cells as Control (water) and Control-losartan cells were very similar in all sets of measurements (Fig. 4, C and G).

We also treated *Fbn1* mutant mice with losartan for an extended period (8 weeks), starting at 2 months after tamoxifen induction (Fig. 4H). The rationale for this timing was to assess whether losartan could restore the satellite cell population or prevent its depletion. This approach also aligned with clinical practice as patients with MFS typically begin losartan treatment after disease onset (*35*). The number of satellite cells in cKO^Homo^ muscles showed a significant degree of rescue (Fig. 4I and fig. S4I). Moreover, in both cKO^Het^ and cKO^Homo^ groups, pSMAD3 levels were restored and were comparable to control groups, indicating potent inactivation of TGFβ signaling in *Fbn1* cKO satellite cells and return to quiescence levels (Fig. 4J). This was further supported by the full reestablishment of projections in the remaining satellite cells across the board (Fig. 4K).

Encouraged by the rescue results on the cellular level, we next evaluated the impact of losartan treatment on muscle architecture and functional performance. As shown in Fig. 4L, losartan treatment resulted in almost complete restoration of muscle morphology, and functional assays using grip strength and treadmill endurance tests demonstrated partial to full recovery of muscle strength and performance (Fig. 4, M and N). By measuring the CSA of the myofibers, we did not observe any statistically significant difference between the size groups and genotypes, but a clear shift in considerable bigger myofibers was observed compared to nontreated mice (fig. S4J).

## DISCUSSION

A central challenge in stem cell biology is to uncover the diverse strategies that stem cells have evolved to preserve their regenerative potential and quiescent state within dynamic and physiologically demanding tissue environments. In satellite cells, intrinsic properties and extrinsic signals, such as transcription factors or factors secreted by neighboring myofibers, contribute to their maintenance. Most of the factors identified to date that regulate satellite cell quiescence act to reinforce quiescence, either by preventing differentiation (Notch signaling), ensuring correct anchoring (Integrinβ1 and Cadherins M/N), or selectively enabling translation of specific mRNAs required for the quiescent state (initiation factor 2 alpha eIF2α) (*3*, *39*–*41*). Some mechanisms, however, function specifically to prevent early activation. For example, CalcR maintains satellite cell quiescence by sensing collagen V in the niche and activating Lats1/2, which phosphorylates and inhibits YAP, thus blocking the transcriptional programs required for activation (*4*, *42*). Similarly, the RTK signaling antagonist Sprouty1 preserves satellite cell quiescence by inhibiting fibroblast growth factor (FGF)–induced ERK signaling, thus preventing their activation (*43*). Our findings reveal a previously unidentified paradigm in stem cell regulation and expand the panel of quiescence mechanisms regulated by Notch signaling (*2*). Here, we show that Notch-driven FBN1 deposition forms a subbasal lamina “quiescence dome,” a protective ECM barrier assembled by the stem cells themselves, that insulates them from mitogenic cues in the environment (fig. S4K). This cell-autonomous niche component complements previously known quiescence regulators and underscores the active role of the stem cells in constructing their own microenvironmental niche.

We also propose that the cell-autonomous function of FBN1 is preserved during tissue regeneration. In resting muscle, satellite cells reside tightly confined between the myofiber and its basal lamina, physically separated from the interstitial space where FBN1 is abundant. This defined anatomical niche is drastically remodeled following injury, with myoblasts, mesenchymal cells and infiltrating immune cells intermingling, and FBN1 remaining abundant or even increased throughout the tissue. Nevertheless, Pax7^+^ progenitors lacking FBN1 fail to self-renew, demonstrating an absolute cell-autonomous requirement for FBN1 production both in quiescent and proliferating progenitors.

Notably, the accepted current model for thoracic aortic diseases such as MFS is that the primary driver is defective adhesion of smooth muscle cells to a compromised ECM, with TGFβ overactivation representing a secondary repair response (*11*). In contrast, we found that inhibition of TGFβ signaling in *Fbn1* cKO satellite cells fully restored their quiescent projections, which are adhesion-dependent structures that sense niche biomechanics (*44*). Restoration of quiescence with robust cytoplasmic projections in satellite cells lacking FBN1 argues against a model in which TGFβ hyperactivation is merely secondary to defective adhesion. Collectively, our analyses of *Fbn1* cKO mice and their response to losartan suggest that FBN1 functions primarily as a signaling regulator rather than a mechanical element of the satellite cell niche.

Beyond the specific context of muscle, because FBN1 is widely expressed in various tissues, a pertinent question is whether similar autocrine matrix–based quiescence mechanisms operate in other stem cell compartments and developmental processes. In the developing retinal vasculature, FBN1 acts at the angiogenic front that regulates endothelial tip/stalk specification by reducing vascular endothelial growth factor (VEGF)–induced Delta-like 4 induction and Notch signaling, largely through its influence on associated proteins like MAGP1 and ADAMTS1 (*45*). Also, in the developing marrow niche, FBN1 modulates TGFβ availability, thereby regulating skeletal stem cell activity (*46*). Notably, although systemic administration of a TGFβ-neutralizing antibody improved bone mass and restored marrow adipogenesis, treatment with losartan did not alleviate bone loss in MFS mice (*47*, *48*). Instead, we found that losartan rescued the depletion of the satellite cell pool and restored both quiescence and muscle function. It would be of great interest to investigate whether the differential responses of various stem cell types reflect distinct niche properties, as well as intrinsic differences between proliferative (emerging) and nonproliferative (quiescent) tissue environments.

Last, our findings provide new insight into the pathophysiology of MFS myopathy. We observed that muscle biopsies from patients with MFS contain activated satellite cells, indicating a compromised stem cell pool in the disease. Encouragingly, the losartan rescue of satellite cell function and muscle performance in our mouse model hints at a therapeutic opportunity.

Therefore, losartan, already in clinical use for preventing aortic aneurysm in MFS, may also alleviate muscle degeneration by normalizing the satellite cell niche signaling environment. Our work thereby positions muscle stem cell dysfunction as a previously underappreciated driver of MFS myopathy.

## MATERIALS AND METHODS

### Mouse strains

The mouse lines used in this study have been previously described and kindly provided as described: *Tg:Pax7-nGFP* [S. Tajbakhsh; (*49*)], [*R26R^stop-NICD-nGFP^* (D. Melton; the Jackson Laboratory, stock 008159; (*50*)], *Pax7-^CreERT2^* [G. Kardon; the Jackson Laboratory, stock 017763; (*51*)], *Pdgfra-^CreERT2^* (*52*), *R26R^td Tomato^* (*53*), and *Fbn1^flox^* [F. Ramirez; (*54*)]. Animals were handled as per French and European Community guidelines, and protocols were approved by the ethics committee at the French Ministry (project no. 20-027 #24357). Mice used were 6 to 12 weeks old.

### Human muscle biopsies

Muscle biopsies were obtained from diagnosed MFS described in (*33*), with protocol number CMO Radboudumc number (CMO-nr.): 2005/311.

### Muscle injury

Mice were anesthetized with isoflurane using the open-drop and nose cone method. Once the mouse was confirmed unresponsive to noxious stimuli (i.e., toe pinch), the lower part of the leg was shaved for better visualization, and the TA muscle was injected with 50 μl of 1.2% BaCl_2_ (Sigma-Aldrich, 202738) using a 28-gauge needle.

### Tissue fixation for immunofluorescence

TA and diaphragm muscles were fixed in 2% paraformaldehyde (PFA)/phosphate-buffered saline (PBS) for 2 hours at 4°C and washed three times with 1X PBS. Diaphragm muscles were then directly subjected in whole-mount staining (see the section “Immunostaining”). TA muscles were immersed in 15% sucrose/PBS overnight, transferred in 30% sucrose/PBS the following day for 4 to 5 hours, embedded in OCT, frozen in liquid-nitrogen-cooled isopentane, and sectioned transversely at 8 μm. Pituitary glands were fixed in 4% PFA/PBS for 12 hours at 4°C, washed three times with 1X PBS, and further processed as TA and diaphragm. Human muscle biopsies were frozen fresh. Once thawed, they were fixed in 4% PFA/PBS overnight at 4°C, washed three times with 1X PBS, embedded in paraffin, and sectioned transversely at 4 μm.

### Single myofiber isolation and siRNA transfection

Individual myofibers were isolated from extensor digitorum longus (EDL) muscles following a modified version of a previously described protocol [Shinin *et al.* (*55*)]. Briefly, EDL muscles were incubated at 37°C in 0.2% collagenase type I (Sigma-Aldrich, C0130) in isolation medium (GlutaMAX DMEM, Thermo Fisher Scientific, 61965069) without antibiotics for 2 hours. Following enzymatic digestion, individual myofibers were released by mechanical dissociation, transferred to serum-coated petri dishes, and transfected with *Fbn1* siRNA [Dharmacon SMARTpool; ON-TARGETplus *Fbn1* (14118) L-045295-01-0020] or scramble siRNA (Dharmacon ON-TARGETplus Non-targeting D-001810-01-20) at a final concentration of 200 nM, using Lipofectamine 2000 (Thermo Fisher Scientific, 11668) in Opti-MEM (Gibco, 31985-070). The following day, the fibers were placed in culture medium [isolation medium with 1% penicillin/streptomycin (GIBCO), 20% fetal bovine serum (FBS), and 1% rat muscle extract], incubated for 72 hours at 37°C/3% O_2_, and fixed in 4% PFA/PBS before immunostaining.

### Isolation, culture, and manipulation of satellite cells

Hindlimb muscles were dissected and enzymatically digested in Dispase A and Collagenase II as previously described [Baghdadi *et al.* (*4*)]. Satellite cells were isolated by FACS (85-μm nozzle) using either GFP or RFP markers depending on the mouse line. Isolated satellite cells were plated on Matrigel-coated (Corning, 354248), 8-chamber slides (Sarstedt, 94.6140.802) in growth medium composed of Dulbecco’s modified Eagle’s medium (DMEM; GIBCO) with 20% FBS, and 1% penicillin/streptomycin (GIBCO), at 37°C. For *Pax7^CreERT2^; R26^stop-NICD-ires-nGFP^*, before FACS, bulk muscle preparations were precultured for 3 days. siRNA transfection of isolated satellite cells was performed as described in the “Single myofiber isolation and siRNA transfection” section. Satellite cells from this mouse line were cultured for 6 days before siRNA. Following siRNA transfection, cells were pulsed with 10 μM EdU (Click-iT PLUS Kit C10640, Life Technologies) for 24 hours before fixation. For reserve cell cultures, isolated satellite cells were cultured for 10 to 14 days [as described by Baghdadi *et al.* (*4*)] and fixed in 4% PFA/PBS for 10 min, followed by three washes in 1X PBS before immunostaining.

### Isolation of FAPs by antibody-based FACS

Hindlimb muscles were isolated and processed as previously described [Baghdadi *et al.* (*4*)]. Bulks were incubated in 2% bovine serum albumin (BSA)/PBS for 15 min on ice and centrifuged for 5 min at 600*g* at 4°C. Bulk pellets were resuspended in 200 μl of antibody mix (antibodies used are listed in table S2) and incubated for 45 min on ice protected from light. Following incubation, cells were centrifuged for 5 min at 600*g* at 4°C, washed once with ice-cold 1X PBS, and resuspended in 0.2% BSA/PBS.

### Immunostaining

After fixation, cells, myofibers, and diaphragms were washed three times with 1X PBS and then permeabilized and incubated in blocking solution containing 5% BSA and 0.25% Triton X-100 in PBS for 60 min at room temperature (RT). Following incubation, the samples were incubated with primary antibodies (table S1) overnight at 4°C. After three washes with 1X PBS, samples were incubated with the corresponding secondary antibodies and Hoechst for 1 hour at RT. EdU staining was performed right after the secondary antibody incubation according to the manufacturer’s instructions (Click-iT PLUS Kit C10640). Laminin staining was performed with a conjugated version of pan-Laminin antibody (NB300-144AG647, Bio-Techne) after secondary antibody incubation for 2 hours at 37°C followed by Hoechst staining and mounting in Fluoromount (00-4958-02, Invitrogen). Tissue sections from TA and pituitary glands were treated the same way as described above with an additional fixation before the blocking step in 4% PFA/PBS.

### Construction of luciferase reporter and luciferase assays

Candidate enhancers of *Fbn1*, *Keratin9*, and *Cadherin15* were amplified by PCR from genomic DNA of C2C12 cells. The enhancers were inserted into the firefly luciferase pGL3-Basic vector (Promega, E1751) upstream of a minimal thymidine kinase promoter and then sequenced. C2C12 cells were transfected using Lipofectamine LTX (Life Technologies, 15338030) and lysed, and the luciferase signal was measured using the Dual-Luciferase Reporter Assay System (Promega, E1910).

For normalization, *Renilla* luciferase (pCMV-Renilla) was transfected at a 1:20 ratio relative to firefly luciferase constructs. The sequences of enhancers are listed in table S3.

### RNA isolation and RT-qPCR

Total RNA was extracted from satellite cells isolated by FACS using the RNA/DNA Microprep kit (Zymo Research, R1051) and reverse transcribed using SuperScript III (Invitrogen, 18080093), as per the manufacturer’s instructions. RT-qPCR was performed using the FastStart Universal SYBR Green Master mix (Roche, 04913914001). Primers used for qPCR analysis are listed in table S4.

### ScRNA-seq data analysis

Publicly available scRNA-seq data from regenerating mouse skeletal muscle were obtained from the study in (*23*). The original dataset was downloaded from the NCBI Gene Expression Omnibus (GEO) under accession number: GSE 138826. All downstream analyses were performed using R (version 4.4.2) and the Seurat package (version 5.3.0) (*56*). Raw count matrices were processed following standard Seurat workflows, including quality control, normalization, dimensionality reduction, clustering, and visualization. Cell type annotation was based on the original metadata provided in the publication and further refined using canonical marker genes. *Pax7*-positive cells were first defined as satellite cells, and this population was extracted using the subset() function in Seurat. The subsetted satellite cells were then reclustered, and clusters characterized by high Pax7 expression were identified and isolated as qSCs. Violin plots of gene expression during muscle regeneration were generated using these qSCs, as shown in Fig. 1F. All custom R scripts and analysis pipelines used in this study are available at the Supplementary Materials (code S1).

### Losartan treatment

Six-week-old mice were administered losartan potassium (Sigma-Aldrich, catalog no. L1299) via drinking water at a concentration of 0.6 g/liter or received placebo (regular water). Drinking bottles were protected from light to preserve drug stability, and the losartan solution was replaced twice weekly for the entire duration of the treatment. Water consumption was monitored throughout the study, and no significant differences were observed between placebo and losartan-treated groups.

### In vivo inhibition TGFβ1, TGFβ2, and TGFβ3

Ten- to 12-week-old mice received a TGFβ1/2/3 neutralizing antibody (1D11; Invitrogen, #MA523795) or an IgG1 isotype control (Invitrogen, #026100) via five intraperitoneal injections, starting 2 weeks after tamoxifen induction, at a dose of 3.75 μg/g body weight. Mice were euthanized 2 weeks after the first injection. Diaphragms and TA muscles were collected and processed as described above.

### TGFβ1 ELISA

To measure active TGFβ1 levels, satellite cells were isolated by FACS using the RFP marker. Isolated satellite cells were plated on Matrigel-coated (Corning, 354248), 96-well plates at 7.5 × 10^3^ cells per well in growth medium, composed of DMEM (GIBCO) with 20% FBS, and 1% penicillin/streptomycin (GIBCO), at 37°C for 4 days. Cells were then starved by replacing growth medium with Opti-MEM (GIBCO) for 3 hours and placed back in growth medium [DMEM with 20% fetal calf serum (FCS)] for 4 hours. Supernatants were collected, and TGFβ1 levels were measured by the Human/Mouse TGFβ1 uncoated ELISA kit (Thermo Fisher Scientific, #88-8350-88) as per the manufacturer’s instructions.

### Muscle performance tests

For the treadmill test, mice were preacclimatized for 5 consecutive days before the test day by placing them on a treadmill (LE87 10RTS, Pan Lab Harvard apparatus) at increasing speed at a rate of 3 cm/s. Mice were left to recover for 2 days before the test day. For the test day, mice ran with a start speed of 8 cm/s and, every 2 min, the speed was increased at a rate of 3 cm/s. The belt slope was set at a 15° inclination, and the electric shock intensity of the resting pad was set at 0.2 mA. Mice ran until exhaustion signs were prominent, i.e., the presence of the mouse to the lower part of the lane, near the shock grid for more than 10 s. Time, speed, and distance ran were noted. For the grip strength test (76-1066, Harvard Apparatus), the mouse was lowered over the grid bar keeping the torso horizontal and allowing only its forepaws to attach to the sensor bar. The mouse was then gently pulled back by its tail ensuring it gripped the horizontal part of the bar, whereas the torso remained horizontal, and the maximal grip strength value was recorded. Each mouse was subjected to the same procedure at least three times per training day with a minimum 5-min interval to allow the mice to recover. The grip test was repeated 2 days later, and the individual measurements were averaged per day per mouse.

### Statistical analysis

For comparison between two groups, unpaired Student’s *t* test was performed to calculate the *P* values. For comparisons between three groups, one-way analysis of variance (ANOVA) was performed. *P* values are indicated in each graph for the respective comparisons. For the correlation between *Fbn1* and *HeyL* expression in satellite cells in scRNA-seq (fig. S1D), ALRA (adaptively-thresholded low rank approximation) was first applied to impute missing expression values based on global cell state trends and mitigate the technical issue of gene dropouts. Monotonic coexpression between the restored gene counts was subsequently assessed using Spearman’s rank correlation test.

## Supplementary Materials

### This PDF file includes:

Figs. S1 to S4 Tables S1 to S4 Code S1
