# Supplementary material for: Muscle stem cells produce a protective Fibrillin-1 matrix to prevent precocious activation
Source: Sci Adv. 2026 Oct 16;12(42):eaeb8190. doi: 10.1126/sciadv.aeb8190 (PMC13674515; doi:10.1126/sciadv.aeb8190)
Supplement: Supplementary file 1 [file sciadv.aeb8190_sm.pdf]

Science Advances
AAAS

Supplementary Materials for

Muscle stem cells produce a protective Fibrillin-1 matrix to prevent precocious activation

Eleni Chrysostomou *et al.*

Corresponding author: Philippos Mourikis, philippos.mourikis@inserm.fr

Sci. Adv. **12**, eaeb8190 (2026)
DOI: 10.1126/sciadv.aeb8190

This PDF file includes:

Figs. S1 to S4
Tables S1 to S4
Code S1

Figure S1

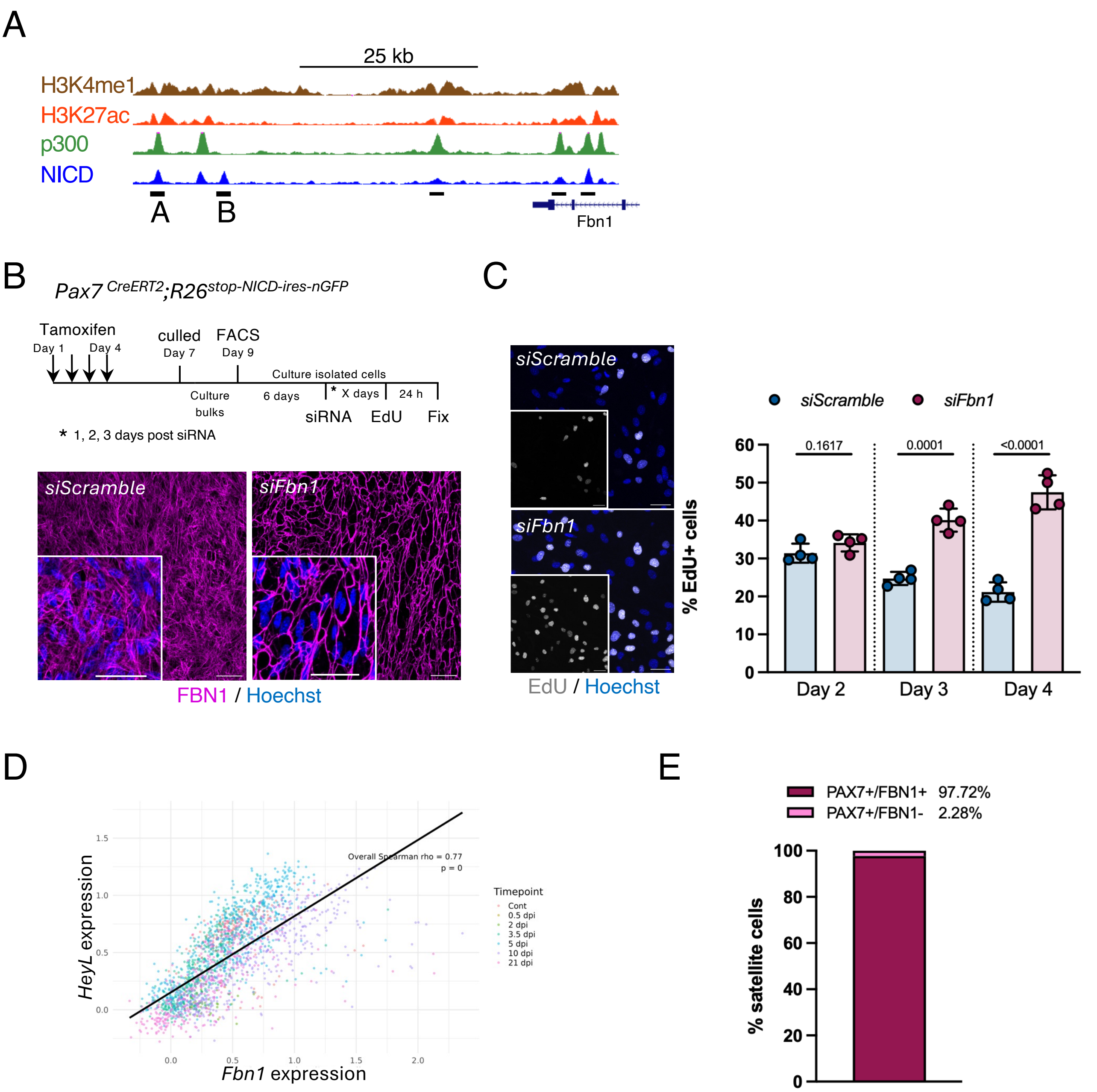
A
25 kb
H3K4me1
H3K27ac
p300
NICD
A
B
Fbn1
B
Pax7 CreERT2;R26stop-NICD-ires-nGFP
Tamoxifen
Day 1
Day 4
culled
Day 7
FACS
Day 9
Culture isolated cells
Culture bulks
6 days
* X days
24 h
siRNA
EdU
Fix
* 1, 2, 3 days post siRNA
siScramble
siFbn1
FBN1 / Hoechst
C
siScramble
siFbn1
EdU / Hoechst
siScramble
siFbn1
% EdU+ cells
0.1617
0.0001
<0.0001
Day 2
Day 3
Day 4
D
Overall Spearman rho = 0.77
p = 0
Timepoint
Cont
0.5 dpi
2 dpi
3.5 dpi
5 dpi
10 dpi
21 dpi
HeyL expression
Fbn1 expression
E
PAX7+/FBN1+ 97.72%
PAX7+/FBN1- 2.28%
% satellite cells

Figure S1: Transcriptional regulation of Fbn1 by Notch signaling.

A. NICD-bound enhancers associated with mouse *Fbn1* loci co-occupied by the histone acetyltransferase p300 and also featuring the hallmark modifications of histone H3 Lys 4 monomethylation (H3K4me1) and/or histone H3 Lys 27 acetylation (H3K27ac); enhancers A and B were cloned, Fig. 1B). **B**. Experimental schematic for *Fbn1* downregulation in FACS-isolated satellite cells. Representative images of si*Scramble* and si*Fbn1* knock-down cultures 4 days post siRNA transfection. **C**. Quantification of EdU^+ cells 2, 3, and 4 days post siRNA, and representative EdU immunofluorescence images at day 4 post siRNA. Unpaired *t-test* ±S.D.; scale bars = 40µm. **D**. Correlation between *Fbn1* and *HeyL* expression in quiescent satellite cells in scRNA-seq. Expression data were retrieved from scRNA-seq described in Oprescu et al., 2020. **E**. Quantification of FBN1 expression relative to PAX7 in cultures from FACS-isolated *Tg:Pax7-nGFP* satellite cells (n=3 mice).

Figure S2

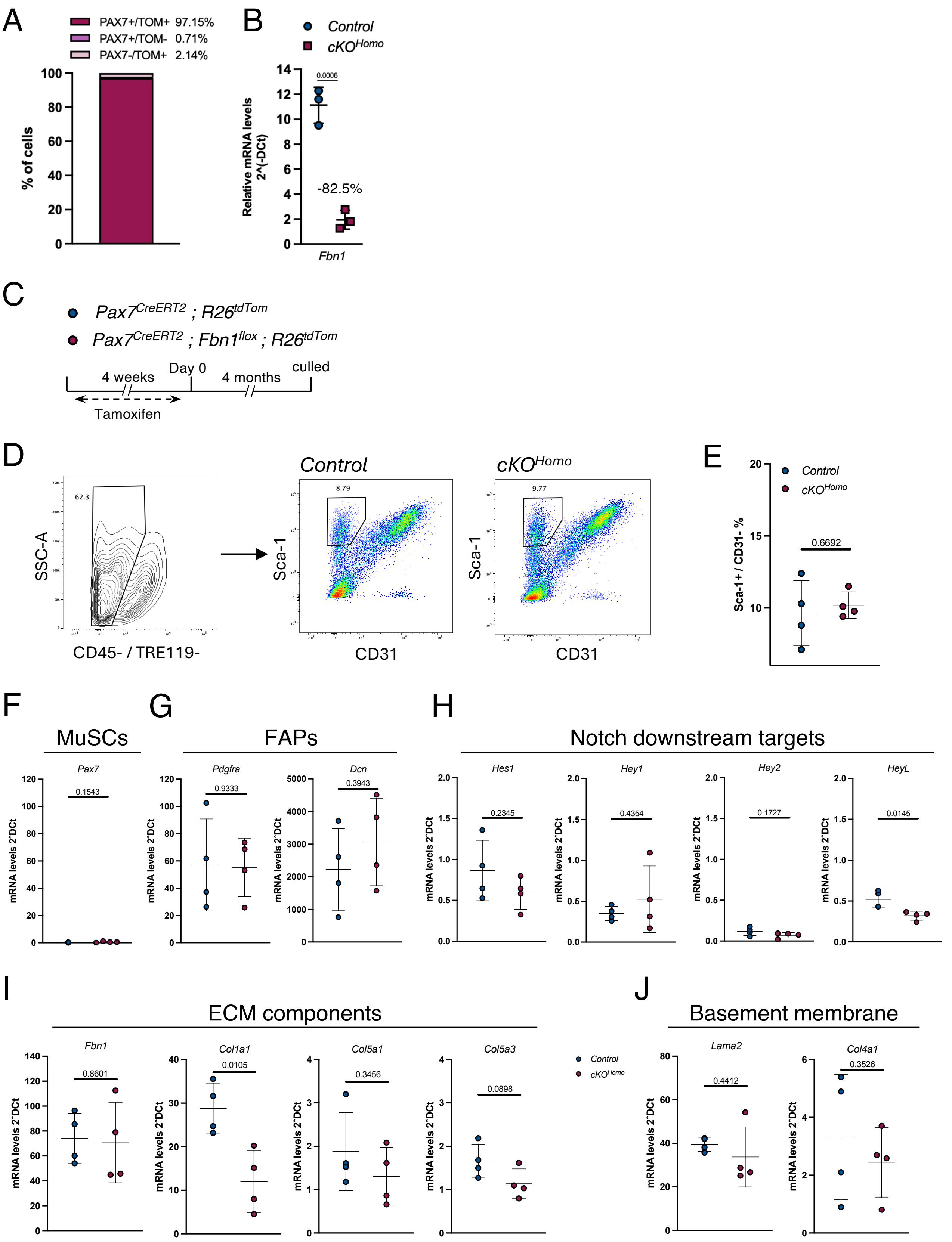

A
PAX7+/TOM+ 97.15%
PAX7+/TOM- 0.71%
PAX7-/TOM+ 2.14%
% of cells
B
Control
cKOHomo
Relative mRNA levels 2^(-DCt)
0.0006
-82.5%
Fbn1
C
Pax7CreERT2 ; R26tdTom
Pax7CreERT2 ; Fbn1flox ; R26tdTom
4 weeks
Day 0
4 months
culled
Tamoxifen
D
SSC-A
62.3
CD45- / TRE119-
Control
8.79
Sca-1
CD31
cKOHomo
9.77
E
Sca-1+ / CD31- %
0.6692
F
MuSCs
Pax7
0.1543
mRNA levels 2^DCt
G
FAPs
Pdgfra
0.9333
Dcn
0.3943
H
Notch downstream targets
Hes1
0.2345
Hey1
0.4354
Hey2
0.1727
HeyL
0.0145
I
ECM components
Fbn1
0.8601
Col1a1
0.0105
Col5a1
0.3456
Col5a3
0.0898
J
Basement membrane
Lama2
0.4412
Col4a1
0.3526

Figure S2 - continued

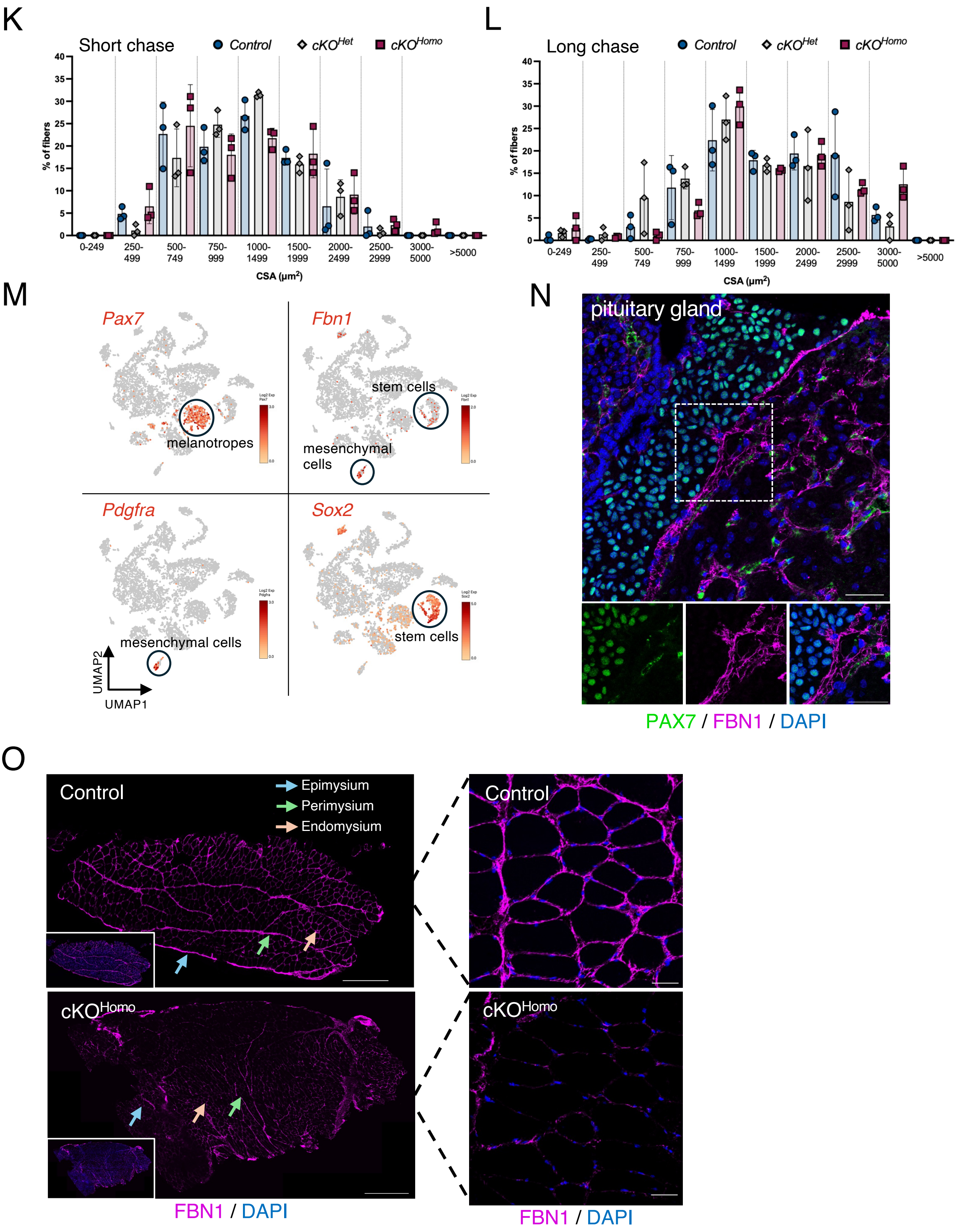

Figure S2: Characterization of Fbn1 deletion in Pax7$^+$ muscle and pituitary gland cells.

A. Percentage of cells expressing Pax7, Tom, or Pax7/Tom from control (*Pax7^{CreERT2};Fbn1$^{+/+}$;R26^{tdTom}*) mice (n=3 mice) based on immunofluorescent staining of TA muscle sections (PAX7$^+$/TOM$^+$ vs PAX7$^+$/TOM$^-$ p=<0.0001; PAX7$^+$/TOM$^+$ vs PAX7$^-$/TOM$^+$ p=<0.0001; PAX7$^+$/TOM$^-$ vs PAX7$^-$/TOM$^+$ p=0.0322). **B.** RT-qPCR analysis of *Fbn1* from TOM$^+$ isolated satellite cells from Control and cKO^{Homo} mice (n=3 mice/genotype) two weeks post tamoxifen treatment. **C.** Experimental scheme for long chase (4 months post tamoxifen) in control (*Pax7^{CreERT2};Fbn1$^{+/+}$;R26^{tdTomato}*) and *Fbn1* homozygous conditional knockout (cKO^{Homo}: *Pax7^{CreERT2};Fbn1$^{fl/fl}$;R26^{tdTomato}*) satellite cells. **D.** Gating strategy for antibody-based cell sorting of FAPs, and **E.** Rercentage of FAPs in control and cKO^{Homo} mice (n=4). **F**. RT-qPCR analysis of *Pax7* (satellite cell marker); **G.** *Pdgfra* and *Dcn* (FAPs markers); **H.** *Hes1*, *Hey1*, *Hey2* and *HeyL* (Notch signalling downstream targets); **I.** *Fbn1*, *Col1a1*, *Col5a1* and *Col5a3* (ECM components); and **J.** *Lama2* and *Col4a1* (Basement membrane components) of Sca-1+ / CD31- FAPs from Control and cKO^{Homo} mice. **K.** Quantification of fiber cross-sectional area (CSA) from the short-chased mice (n=3 mice/genotype). **L.** Quantification of CSA from the long-chased cohort (n=3 mice/genotype). **M.** UMAP of single-cell RNAseq from pituitary gland cells indicating the expression of *Pax7*, *Fbn1*, *Pdgfra* and *Sox2* (24). **N.** Immunostaining of pituitary gland sections from wild-type mice. PAX7$^+$ cells in the intermediate lobe do not express FBN1 (magenta). **O.** FBN1 decrease in *Fbn1* cKO^{Homo} in FAPs (*Pdgfra^{CreERT2};Fbn1$^{fl/fl}$;R26^{tdTomato}*) compared to Control (*Pdgfra^{CreERT2};Fbn1$^{+/+}$;R26^{tdTomato}*) TA muscle sections [n=3 mice/genotype; scale bar = 200 µm (left panels), 40 µm (right panels)]. Unpaired *t-test* ±S.D (B; E-J); One-way ANOVA ±S.D. (K-L); scale bars = 40 µm.

Figure S3

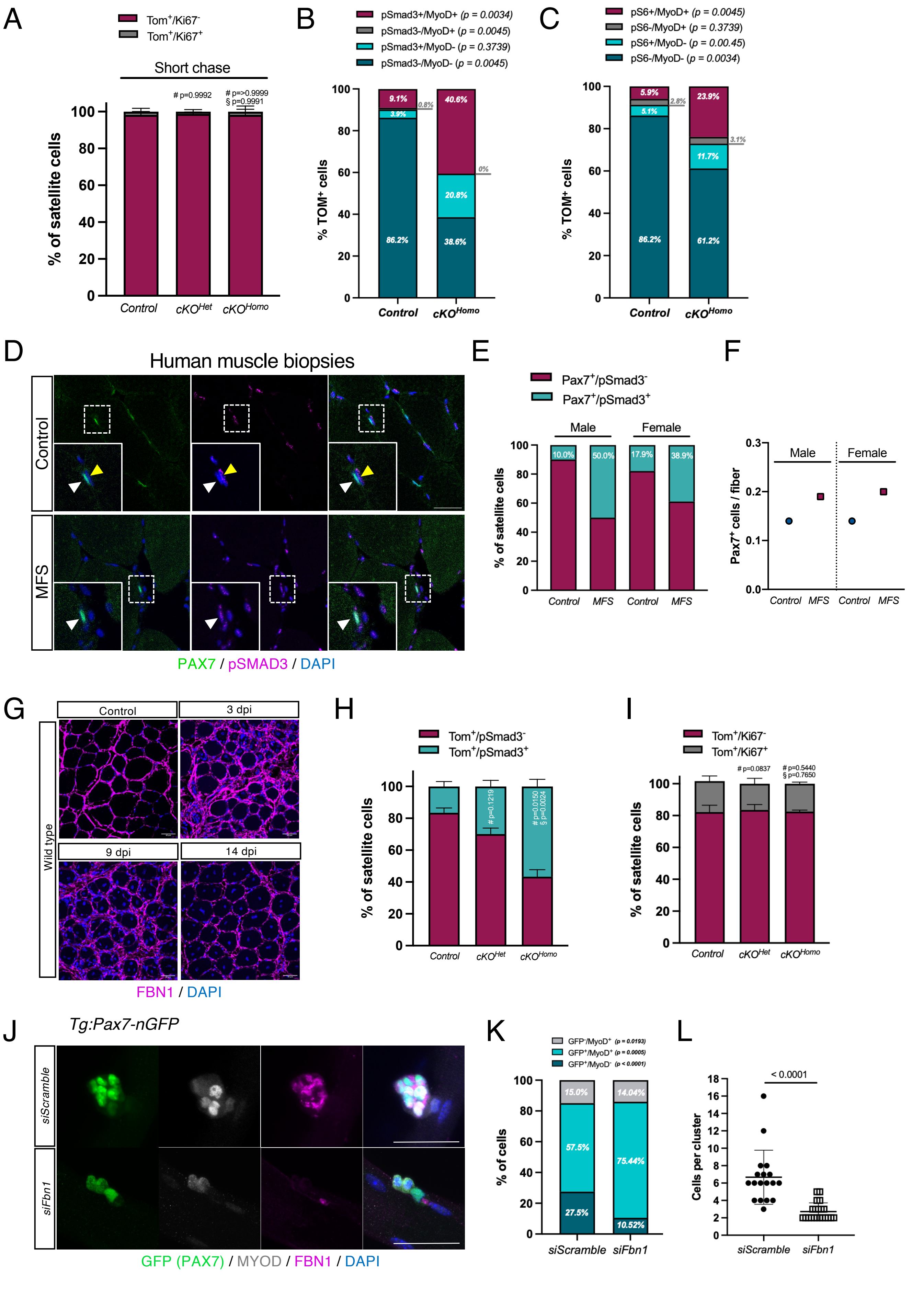

A
Tom+/Ki67-
Tom+/Ki67+
Short chase
% of satellite cells
# p=0.9992
# p=>0.9999
§ p=0.9991
Control
cKOHet
cKOHomo
B
pSmad3+/MyoD+ (p = 0.0034)
pSmad3-/MyoD+ (p = 0.0045)
pSmad3+/MyoD- (p = 0.3739)
pSmad3-/MyoD- (p = 0.0045)
% TOM+ cells
9.1%
0.8%
3.9%
86.2%
40.6%
0%
20.8%
38.6%
Control
cKOHomo
C
pS6+/MyoD+ (p = 0.0045)
pS6-/MyoD+ (p = 0.3739)
pS6+/MyoD- (p = 0.00.45)
pS6-/MyoD- (p = 0.0034)
% TOM+ cells
5.9%
2.8%
5.1%
86.2%
23.9%
3.1%
11.7%
61.2%
Control
cKOHomo
D
Human muscle biopsies
Control
MFS
PAX7 / pSMAD3 / DAPI
E
Pax7+/pSmad3-
Pax7+/pSmad3+
Male
Female
% of satellite cells
10.0%
50.0%
17.9%
38.9%
Control
MFS
Control
MFS
F
Male
Female
Pax7+ cells / fiber
Control
MFS
Control
MFS
G
Control
3 dpi
9 dpi
14 dpi
Wild type
FBN1 / DAPI
H
Tom+/pSmad3-
Tom+/pSmad3+
% of satellite cells
# p=0.1219
# p=0.0150
§ p=0.0024
Control
cKOHet
cKOHomo
I
Tom+/Ki67-
Tom+/Ki67+
% of satellite cells
# p=0.0837
# p=0.5440
§ p=0.7650
Control
cKOHet
cKOHomo
J
Tg:Pax7-nGFP
siScramble
siFbn1
GFP (PAX7) / MYOD / FBN1 / DAPI
K
GFP-/MyoD+ (p = 0.0193)
GFP+/MyoD+ (p = 0.0005)
GFP+/MyoD- (p < 0.0001)
% of cells
15.0%
57.5%
27.5%
14.04%
75.44%
10.52%
siScramble
siFbn1
L
< 0.0001
Cells per cluster
siScramble
siFbn1

Figure S3: FBN1 knockdown impairs self-renewal in vitro and satellite cell recovery during regeneration.

A. Quantification of $Tom^+/Ki67^\pm$ satellite cells in Control, Fbn1 cKO^{Het} and Fbn1 cKO^{Homo} mice from short chase after *Fbn1* deletion (n = 3 mice/genotype). **B.** Quantification of $pSmad3^\pm/MyoD^\pm$, and **C.** $pS6^\pm/MyoD^\pm$ of Tom^+ satellite cells in Control and *Fbn1* short-chased mutant satellite cells (n=3 mice/genotype). **D**. Immunostaining of *Vastus lateralis* muscles from age- and sex-matched control and MFS patients (white arrowheads indicate satellite cells; yellow arrowheads point pSMAD3+/PAX7- cell). **E.** Quantification of $PAX7^+/pSMAD3^\pm$ satellite cells. One-way ANOVA ±S.D. **F.** Quantification of PAX7+ cells on human muscle biopsies. **G.** Immunostaining of TA muscle sections from wild-type mice at 0 days (control), 3, 9 and 14 days post TA injury. **H.** $Tom^+/pSMAD3^\pm$, and **I.** $Tom^+/Ki67^\pm$ satellite cells in control and *Fbn1* cKO mice (n=3 mice per genotype) 14 days post injury. **J.** Immunostaining of 72hr-cultured individual myofibers from *Tg:Pax7-nGFP* mice transfected with *siScramble* (top panel) or *siFbn1* (bottom panel). **K.** Quantification of $GFP^+/MyoD^-$, $GFP^+/MyoD^+$, and $GFP^-/MyoD^+$ cells from the resulted clusters (n=3 per siRNA), and **L.** of cluster sizes. One-way ANOVA ±S.D. (A-C, H-I); # compared to Control; § compared to cKO^{Het} ; unpaired *t-test* ±S.D (L); Scale bars = 40 µm.

Figure S4

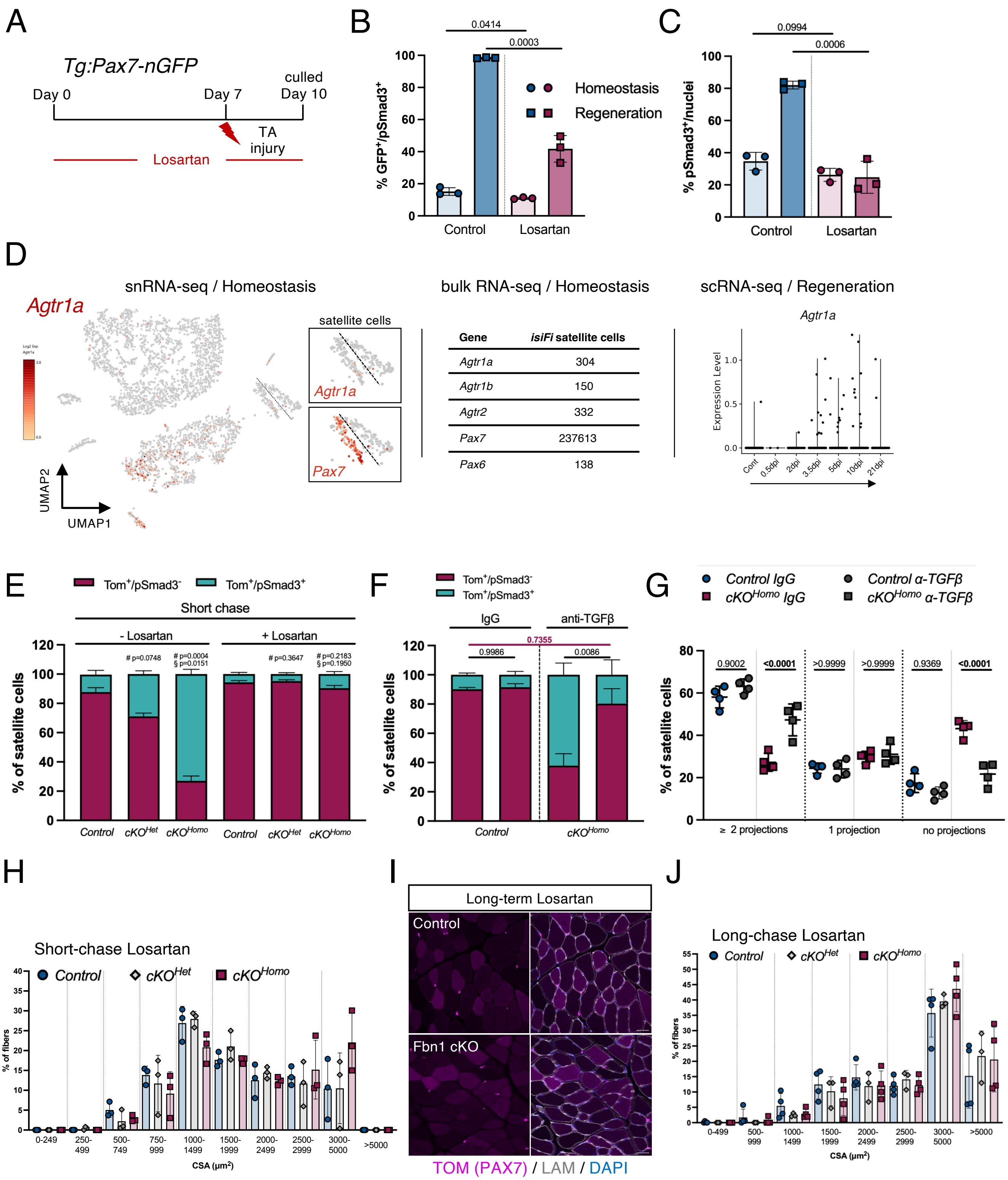
A
Tg:Pax7-nGFP
Day 0
Day 7
culled
Day 10
TA
injury
Losartan
B
% GFP+/pSmad3+
0.0414
0.0003
Homeostasis
Regeneration
Control
Losartan
C
% pSmad3+/nuclei
0.0994
0.0006
Control
Losartan
D
Agtr1a
snRNA-seq / Homeostasis
satellite cells
Agtr1a
Pax7
UMAP2
UMAP1
bulk RNA-seq / Homeostasis
Gene | isiFi satellite cells
Agtr1a | 304
Agtr1b | 150
Agtr2 | 332
Pax7 | 237613
Pax6 | 138
scRNA-seq / Regeneration
Agtr1a
Expression Level
Cont
0.5dpi
2dpi
3.5dpi
5dpi
10dpi
21dpi
E
Tom+/pSmad3-
Tom+/pSmad3+
Short chase
- Losartan
+ Losartan
# p=0.0748
# p=0.0004
§ p=0.0151
# p=0.3647
# p=0.2183
§ p=0.1950
% of satellite cells
Control
cKOHet
cKOHomo
F
Tom+/pSmad3-
Tom+/pSmad3+
IgG
anti-TGFβ
0.7355
0.9986
0.0086
% of satellite cells
Control
cKOHomo
G
Control IgG
cKOHomo IgG
Control α-TGFβ
cKOHomo α-TGFβ
0.9002
<0.0001
>0.9999
>0.9999
0.9369
<0.0001
% of satellite cells
≥ 2 projections
1 projection
no projections
H
Short-chase Losartan
Control
cKOHet
cKOHomo
% of fibers
0-249
250-499
500-749
750-999
1000-1499
1500-1999
2000-2499
2500-2999
3000-5000
>5000
CSA (µm2)
I
Long-term Losartan
Control
Fbn1 cKO
TOM (PAX7) / LAM / DAPI
J
Long-chase Losartan
Control
cKOHet
cKOHomo
% of fibers
0-499
500-999
1000-1499
1500-1999
2000-2499
2500-2999
3000-5000
>5000
CSA (µm2)

Supp. Figure 4 continued

K

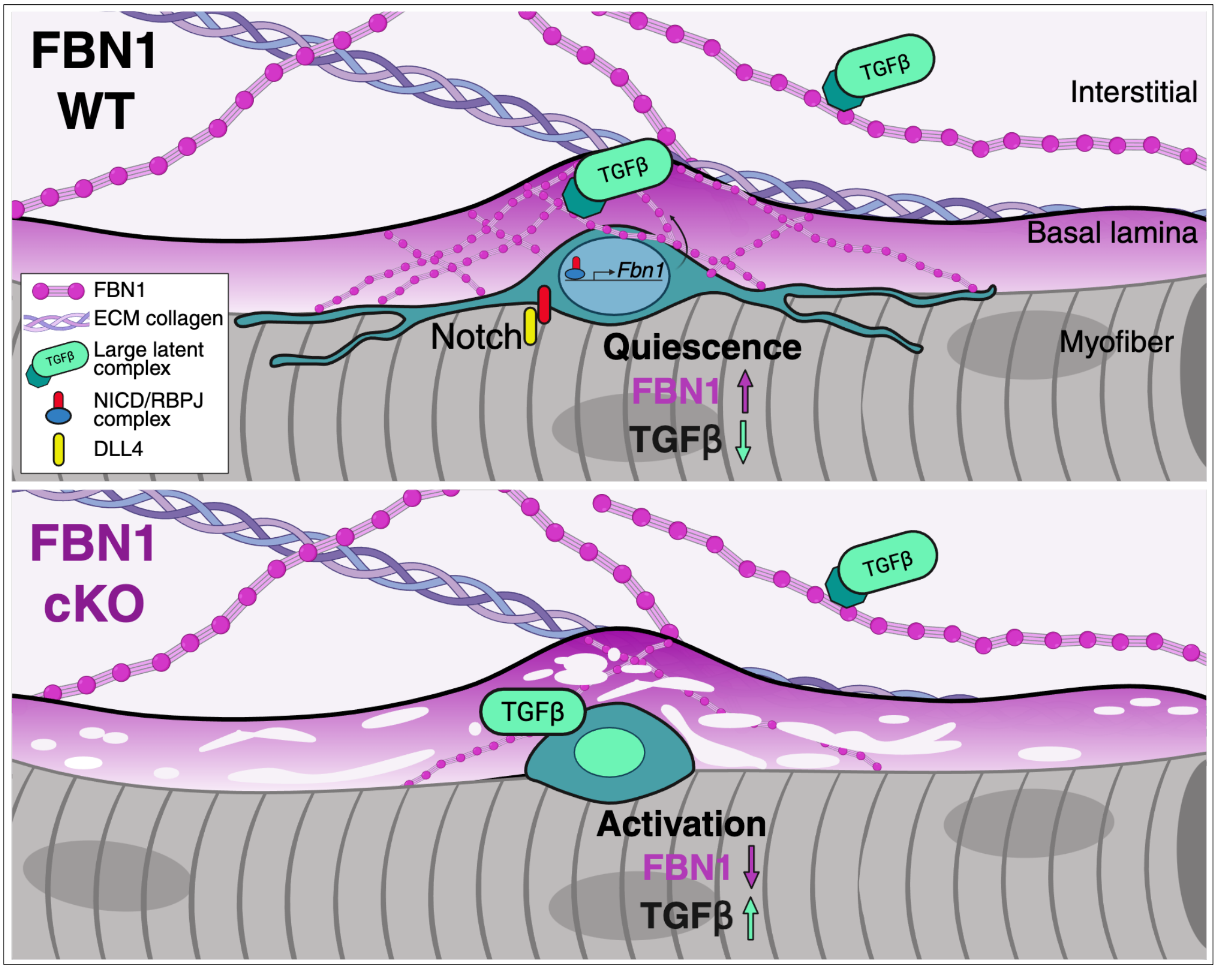

Figure S4: Angiotensin II type 1 receptor antagonist restores quiescence of Fbn1-deficient satellite cells and prevents muscle performance decline.

A. Experimental scheme of losartan administration to *Tg:Pax7-nGFP* mice. **B.** Quantification of pSMAD3^{+} satellite cells (GFP^{+}) and **C.** total pSMAD3^{+} nuclei. **D.** Expression of *Agtr1a* in snRNAseq from intact TA muscle (18), in bulk RNAseq on *in situ* fixed satellite cells from hind limb muscles (17), and scRNAseq from injured TA during regeneration (21). In the bulk RNAseq table, *Pax7* is shown as positive control and *Pax6*, which is not expresses in muscle, as background baseline of the reads. **E.** Tom^{+}/pSmad3$^{\pm}$ in homeostatic TA muscles (n = 3-6 mice/genotype) with and without exposure to losartan (n = 3 mice/genotype per treatment). **F.** Tom^{+}/pSmad3$^{\pm}$ in homeostatic TA muscles in Fbn1 cKO and control mice after IgG or anti-TGFβ administration (n = 3 mice/genotype per treatment). **G.** Quantification of satellite cell projections in mice treated with IgG or anti-TGFβ (n = 3 mice/genotype per treatment). **H.** Quantification of fiber cross-sectional area (CSA) from the short-term administration of losartan. **I.** Immunostaining of TA muscle sections from Control and *Fbn1* cKO TA muscles from cohorts with and without losartan (long-term exposure). and **J.** Quantification of fiber cross-sectional area (CSA) from the long-term losartan exposure in *Fbn1*-cKO cohorts (n = 3 mice per genotype). **K**. Proposed model of Notch-FBN1-TGFβ axis in the maintenance of skeletal muscle stem cell quiescence. Graphic created in BioRender. Chrysostomou, E. (2026) https://BioRender.com/8eevndu. Unpaired *t-test* ±S.D (B, C); one-way ANOVA ±S.D. (E, F-H; J); scale bars = 40µm.

Supplementary tables

Table S1: List of primary antibodies.

Antibody	Reference	Dilution
GFP	Abcam #13970	1/1000
RFP	Chromotek #CR-5F8	1/250
Fbn1	Shi et al. (20)	1/1500
Pax7	Santa Cruz #sc-81648	1/200
pSmad3	Abcam #ab52903	1/200
Ki67	Abcam #ab16667	1/200
Laminin conjugated	Biotechne #NB300-144AF647	1/200
MyoD (clone 5F11)	Active Motif #39991	1/200
P-S6 (S235/236)	Cell Signalling #2211S	1/200

s

Table S2: List of antibodies used for cytometry.

**Antibody**	**Reference**	**Dilution**
TER119 PE-Cy7	BD #557853	1.75/100
CD45 PE-Cy7	BD #552848	1.75/100
Ly-6A (Sca-1) FITC	BD #553335	1.75/100
CD31 APC	BD #551262	1.33/100

Table S3: Coordinates of the Fbn1-associated enhancers (mouse) used in the luciferase assays.

**Enhancer**	**Chromosome**	**Start-End**	**Size (bp)**
Fbn1A	Chr2	125,101,224 - 125,101,733	509
Fbn1B	Chr2	125,105,576 - 125,106,100	524

Table S4: List of primers used for RT-qPCR (mouse).

**Gene**	**Forward primer (5'>3')**	**Reverse primer (5'>3')**
Fbn1	TGGGTTCCAACTGGCATCAG	ATCCGTGTTCACACAGCGTC
Pax7	GACAAAGGGAACCGTCTGGAT	TGTGAACGTGGTCCGACTG
Pdgfra	TCGAAGGCAGGCACATTTACA	CTAAGGAATCGGTCATCCCGAG
Dcn	CTATGTGCCCCTACCGATGC	TGTCGGGTGGAAAATCCCAG
Hes1	GGCCGAAAGGACGTAAGACT	GTTGGACCGGTGCTAAACCA
Hey1	CACCTGAAAATGCTGCACAC	ATGCTCAGATAACGGGCAAC
Hey2	TTGAAGATGCTCCAGGCTAC	AGCACTCTCGGAATCCAATG
HeyL	GTCTTGCAGATGACCGTGGA	CTCGGGCATCAAAGAACCCT
Col1a1	CATTGTGTATGCAGCTGACTTC	CGCAAAGAGTCTACATGTCTAGG

Col4a1	TGCAACGGTACAAAGGGAGAG	TGGCCGAGAATTTCACCAGG
Col5a1	GCTACTCCTGTTCCTGCTGC	TGAGGGCAAATTGTGAAAATC
Col5a3	CCGGAGACTGGATCAGCTT	GCTTCCAGTACGTCCACAGG
Lama2	ACAGGTGCTGTCCAGGATTC	CCGTGACAATTGCATGCTTCA
Tbp	ATCCCAAGCGATTTGCTG	CCTGTGCACACCATTTTTCC

Supplementary code S1

This code was implemented in R using the Seurat package to perform data loading, integration, preprocessing, visualization, and subclustering of single-cell RNA sequencing data.

```
library(Seurat) #Version-5.3.0
library(dplyr) #Version-1.1.4
library(ggplot2) #Version-3.5.2

########LoadTheData###
base_dir <- "PATH"
# List of SRR project IDs
srr_list <- paste0("SRR102754", 13:19)
# List to store Seurat objects
seurat_objects <- list()
# Create Seurat objects for each SRR project
for (srr in srr_list) {
  data_dir <- file.path(base_dir, paste0(srr, "PATH"))
  mtx_file <- file.path(data_dir, "matrix.mtx.gz")
  barcodes_file <- file.path(data_dir, "barcodes.tsv.gz")
  features_file <- file.path(data_dir, "features.tsv.gz")
  count <- ReadMtx(mtx = mtx_file, cells = barcodes_file, features = features_file)
  seurat_object <- CreateSeuratObject(counts = count, project = srr)
  seurat_objects[[srr]] <- seurat_object
}
#
seurat_obj_cont <- seurat_objects[["SRR10275413"]]
seurat_obj_0.5dpi <- seurat_objects[["SRR10275414"]]
seurat_obj_2dpi <- seurat_objects[["SRR10275415"]]
seurat_obj_3.5dpi <- seurat_objects[["SRR10275416"]]
seurat_obj_5dpi <- seurat_objects[["SRR10275417"]]
seurat_obj_10dpi <- seurat_objects[["SRR10275418"]]
seurat_obj_21dpi <- seurat_objects[["SRR10275419"]]

#####Integration#####
seurat_list <- list(
  seurat_obj_cont,
  seurat_obj_0.5dpi,
  seurat_obj_2dpi,
  seurat_obj_3.5dpi,
  seurat_obj_5dpi,
  seurat_obj_10dpi,
  seurat_obj_21dpi
)

#####labeling#####
timepoints <- c("cont", "0.5dpi", "2dpi", "3.5dpi", "5dpi", "10dpi", "21dpi")
# Merge Seurat objects using merge function
merged_seurat <- merge(
  x = seurat_objects[[1]],
  y = seurat_objects[2:length(seurat_objects)],
  add.cell.ids = timepoints,
  project = "merged"
)
merged_seurat[["percent.mt"]] <- PercentageFeatureSet(merged_seurat, pattern =
"^mt-")
```

```
merged_seurat <- subset(merged_seurat, subset = nFeature_RNA > 200 &
nFeature_RNA < 8000 & percent.mt < 5)
merged_seurat <- NormalizeData(merged_seurat)
merged_seurat <- FindVariableFeatures(merged_seurat)
merged_seurat <- ScaleData(merged_seurat)
merged_seurat <- RunPCA(merged_seurat)
merged_seurat <- RunUMAP(merged_seurat, dims = 1:30)
merged_seurat <- FindNeighbors(merged_seurat, dims = 1:10)
merged_seurat <- FindClusters(merged_seurat, resolution = 0.15)
DimPlot(merged_seurat, reduction = "umap", group.by = "seurat_clusters",label = TRUE)
+
  ggtitle("UMAP Colored by Clusters")
 ggsave('Integrated_umap.png', width=15, height=15)
DimPlot(merged_seurat, reduction = "umap", group.by = "orig.ident") +
  ggtitle("Integrated UMAP")
DimPlot(merged_seurat, reduction = "umap", split.by = "orig.ident", ncol = 2) +
  ggtitle("UMAP Split by Timepoint")

####Targets-genes####
 genes_to_plot <- c("Pax7", "Fbn1", "Heyl", "Hey1", "Hes1", "Notch3",
"Myod1","Mki67","Calcr","Agtr1a","Agtr1b","Agtr1")
 plot_titles <- c("Pax7", "Fbn1", "HeyL", "Hey1", "Hes1", "Notch3",
"Myod1","Mki67","CalcR","Agtr1a","Agtr1b","Agtr1")
 display_names <- c("Pax7", "Fbn1", "HeyL", "Hey1", "Hes1", "Notch3",
"Myod1","Mki67","CalcR","Agtr1a","Agtr1b","Agtr1")
 # re-order
 ordered_labels <- c("Cont", "0.5dpi", "2dpi", "3.5dpi", "5dpi", "10dpi", "21dpi")
 # Vlnplot-for-each
 for (i in seq_along(genes_to_plot)) {
  gene <- genes_to_plot[i]
  disp <- display_names[i]
  expr_data <- FetchData(merged_seurat, vars = gene, slot = "data")
  cells_keep <- rownames(expr_data)[expr_data[[gene]] > 0]
  # subset
  tmp <- subset(merged_seurat, cells = cells_keep)
  # Vlnplot
  p <- VlnPlot(
   tmp,
   features = gene,
   group.by = "orig.ident",
   slot = "data",
   pt.size = 0.5
  ) +
   scale_x_discrete(limits = ordered_labels) +
   ggtitle(bquote(italic(.(disp)))) +
   theme(
    axis.text.x  = element_text(angle = 45, hjust = 1),
    plot.title   = element_text(size = 14, face = "italic", hjust = 0.5),
    plot.margin  = margin(5, 5, 5, 5), legend.position = 'none'
   )
  # Save
  file_name <- paste0("Vln_", gene, ".png")
  ggsave(
   filename = file_name,
   plot     = p,
   width    = 5,
   height   = 5,
   dpi      = 300
```

```
  )
 }

#####Subclustering#####
  seurat_subset <- subset(merged_seurat, idents = c("Pax7-enriched-cluster"))  # select
cluster
  seurat_subset <- NormalizeData(seurat_subset)
  seurat_subset <- FindVariableFeatures(seurat_subset, selection.method = "vst",
nfeatures = 2000)
  seurat_subset <- ScaleData(seurat_subset, features = rownames(seurat_subset))
  seurat_subset <- RunPCA(seurat_subset)
  seurat_subset <- FindNeighbors(seurat_subset, dims = 1:10)
  seurat_subset <- FindClusters(seurat_subset, resolution = 0.25)
  seurat_subset <- RunUMAP(seurat_subset, dims = 1:10)

  # Integrated MuSC-UMAP_orig.ident
  p1 <- DimPlot(seurat_subset, reduction = "umap", group.by = "orig.ident") +
    ggtitle("Integrated MuSC-UMAP")
  print(p1)
  ggsave("Integrated MuSC-UMAP_orig.ident.png", plot = p1, width = 6, height = 6, dpi =
300)
  # MuSC-UMAP Colored by Clusters
  p2 <- DimPlot(seurat_subset, reduction = "umap", group.by = "seurat_clusters", label =
TRUE) +
    ggtitle("MuSC-UMAP Colored by Clusters")
  print(p2)
  ggsave("MuSC-UMAP Colored by Clusters_by_clusters.png", plot = p2, width = 6, height
= 6, dpi = 300)
  VlnPlot(seurat_subset, features =
c("Pax7","Hs6st3","Calcr","Myod1","Myf5","Myog","Ctnnb1","Zbtb18","Mycl","Scx","Mef2a","
Hes6",
"E2f8","Klf5","Id1","Tead4","Piezo1","Piezo2","Mymx","Acta1","Cdkn1c","Myh1","Acta1"))
  ggsave('MuSC-myogenic_vlnplot.png', width=15, height=15)
  FeaturePlot(seurat_subset, features =
c("Pax7","Hs6st3","Calcr","Myod1","Myf5","Myog","Ctnnb1","Zbtb18","Mycl","Scx","Mef2a","
Hes6","E2f8","Klf5","Id1","Tead4","Piezo1","Piezo2","Mymx","Acta1","Cdkn1c","Myh1","Acta1"
))
  ggsave('MuSC-myogenic_umap.png', width=15, height=15)

#####Subclustering-MuSC-> qMuSCandactMuSC#####
  qMuSC <- subset(seurat_subset, idents = c(0, 1, 2, 5))
  actMuSC <- subset(seurat_subset, idents = c(3, 4))
  genes_to_plot <- c("Pax7", "Fbn1", "Heyl", "Hey1", "Hes1", "Notch3",
"Myod1","Mki67","Calcr","Agtr1a")
  display_names <- c("Pax7", "Fbn1", "HeyL", "Hey1", "Hes1", "Notch3",
"Myod1","Mki67","CalcR","Agtr1a")
  ordered_labels <- c("Cont", "0.5dpi", "2dpi", "3.5dpi", "5dpi", "10dpi", "21dpi")
  object_list <- list(qMuSC = qMuSC, actMuSC = actMuSC)
  #
  for (obj_name in names(object_list)) {
    obj <- object_list[[obj_name]]
    for (i in seq_along(genes_to_plot)) {
      gene <- genes_to_plot[i]
      disp <- display_names[i]
      # VlnPlot
      p <- VlnPlot(
        obj,
        features = gene,
```

```
      group.by = "orig.ident",
      slot = "data",
      pt.size = 1
    ) +
      scale_x_discrete(limits = ordered_labels) +
      ggtitle(bquote(italic(.(disp)))) +
      theme(
        axis.text.x = element_text(angle = 45, hjust = 1, family= "Helvetica"),
        plot.title = element_text(size = 18,face = "bold.italic", hjust = 0.5, family=
"Helvetica"),
        plot.margin = margin(5, 5, 5, 5),
        legend.position = 'none'
      )
    # Save
    file_name <- paste0(obj_name, "-Vln_", gene, ".png")
    ggsave(
      filename = file_name,
      plot = p,
      width = 4,
      height = 4,
      dpi = 300
    )
  }
 }
```
